# Age-related molecular and functional remodeling of the choroid plexus in the gray mouse lemur

**DOI:** 10.1371/journal.pone.0357233

**Published:** 2026-08-31

**Authors:** Florent Amiot, Antxon Etchebest, Bijou Andriambelo, Joannie St-Germain, Mariano Avino, Mélanie Plourde, Benoit Laurent, Fabien Pifferi

**Affiliations:** 1 Research Center on Aging, Centre Intégré Universitaire de Santé et Services Sociaux de l’Estrie-Centre Hospitalier Universitaire de Sherbrooke, Sherbrooke, Québec, Canada; 2 Department of Biochemistry and Functional Genomics, Faculty of Medicine and Health Sciences, Université de Sherbrooke, Sherbrooke, Québec, Canada; 3 UMR CNRS MNHN 7179 MECADEV - Mécanismes Adaptatifs et Évolution, Brunoy, France; 4 Department of Pharmacology-Physiology, Faculty of Medicine and Health Sciences, Université de Sherbrooke, Sherbrooke, Québec, Canada; 5 Bio-informatic platform, Department of Biochemistry and Functional Genomics, Faculty of Medicine and Health Sciences, Université de Sherbrooke, Sherbrooke, Québec, Canada; 6 Department of Medicine, Faculty of Medicine and Health Sciences, Université de Sherbrooke, Sherbrooke, Québec, Canada; 7 Institut sur la Nutrition et les Aliments Fonctionnels, Université Laval, Québec City, Québec, Canada; Namik Kemal University: Tekirdag Namik Kemal Universitesi, TÜRKIYE

## Abstract

Aging is accompanied by complex alterations in brain structure and functions, with growing evidence implicating the choroid plexus (CP) as a key regulator of cognitive and motor function. Here, we investigated age-related changes in the CP of the gray mouse lemur (*Microcebus murinus*), a non-human primate model that recapitulates human brain aging features. Lemurs of different ages underwent behavioral testing, followed by transcriptomic profiling and immunofluorescence analyses of lateral ventricle CP tissue. Behavioral assessments revealed age-related declines in motor coordination and exploratory drive, whereas working memory and visual discrimination remained preserved. Histological analysis showed no significant structural alterations in CP architecture. Transcriptomic profiling identified 1,519 upregulated and 1,682 downregulated genes with aging, highlighting increased interindividual heterogeneity, upregulation of immune- and transport-related pathways, and downregulation of signaling and intercellular communication processes. Functionally, AQP1 protein expression decreased with age without changes in mRNA levels, suggesting post-transcriptional regulation, whereas NKCC1 and TTR expression were largely maintained. Notably, reduced AQP1 expression correlated with age-related motor decline. These findings support a model in which aging mouse lemur CP undergoes functional reorganization and selective vulnerability rather than by generalized structural or functional deterioration. Our results highlight a primate-like aging profile of the CP, providing insights into mechanisms by which CP dysfunction may contribute to age-related motor decline and altered brain homeostasis.

## Introduction

Brain aging is a complex, multifactorial process involving structural, functional, and molecular alterations that progressively impair cognition and motor functions. While many studies focused on neuronal and synaptic alterations during aging, increasing evidence indicate that the age-related dysfunction of brain barriers and cerebrospinal fluid (**CSF**) dynamics plays a critical role in maintaining brain homeostasis and cognitive function [1,2]. Among these brain barriers, the choroid plexus (**CP**), an epithelial structure within the brain ventricles responsible for CSF production, has emerged as a major regulator of brain aging [3].

The CP contributes to brain function through multiple mechanisms, including CSF production, regulation of nutrient, waste clearance, immune surveillance, and secretion of trophic and signaling factors that influence neural plasticity [4–6]. Aging is associated with profound molecular and structural changes in the CP, such as epithelial cell atrophy, thickening of the basal membrane, alteration of tight junction integrity, increased inflammation, and changes in secretory profiles [7–9]. Specifically, the aging CP displays downregulation of genes involved in barrier integrity, and CSF production. For instance, the reduced expression of the membrane water channel protein aquaporin-1 (**AQP1**) and the Na⁺-K⁺-2Cl^−^ cotransporter 1 (**NKCC1**) impairs CSF turnover and clearance of brain metabolites [10,11]. A decline in the expression of genes coding proteins secreted into the CSF, like the transthyretin (**TTR**) protein, has also been reported in the aging CP [12]. During aging, the CP undergoes a progressive shift toward a pro-inflammatory state that is reflected in marked changes in gene expression [13,14]. Transcriptomic studies have shown increased expression of immune- and interferon-related genes, including cytokines, and components of antigen presentation pathways. This age-associated CP inflammation alters CSF composition and promotes the entry of peripheral immune signals into the brain to contribute to chronic neuroinflammation [14]. It also disrupts brain homeostasis by reducing the brain repair and waste clearance [15–17].

Recent studies in rodents and humans suggest that age-dependent CP dysfunction may directly influence learning and memory, in part through the modulation of adult neurogenesis [14,15]. In this context, structural and transcriptional alterations of the CP may act as early markers or mechanistic drivers of cognitive decline. However, most of these insights originate from rodent models, which undergo rapid aging and differ substantially from humans in brain organization, immune regulation, and lifespan. Although highly informative, such models may not fully capture the complexity and heterogeneity of CP aging observed in humans. It remains unclear whether similar alterations occur during CP aging in primates, and whether age-related changes in the CP are associated with cognitive performance. Addressing these questions is essential for a deeper understanding of normal brain aging and may provide important insights into mechanisms underlying vulnerability to neurodegenerative diseases.

The gray mouse lemur (*Microcebus murinus*), a small nocturnal non-human primate endemic to Madagascar, represents a unique and powerful model for studying brain aging. With a median lifespan of about 6 years in captivity, mouse lemurs exhibit spontaneous age-related cognitive decline, and neuropathological features reminiscent of human aging, including cerebral atrophy and amyloid deposition [18–20]. Some studies highlighted the mouse lemur value in aging research, particularly for exploring interindividual variability and cognitive decline trajectories that mirror those seen in humans [19,21]. Importantly, their relatively short lifespan and primate-specific brain organization position them as a valuable translational model bridging the gap between rodent and human studies on aging [22,23]. Despite growing interest in the mouse lemur as a model of cognitive aging, the status of the CP and its potential contribution to age-associated cognitive impairments in this species remain largely unexplored [24,25].

In the present study, we investigated the structural, transcriptional, and functional changes occurring in the CP of the mouse lemur during aging. Our goal was to elucidate the role of CP aging in non-human primate brain function and to identify potential mechanisms linking barrier dysfunction to cognitive impairment. We hypothesized that aging in the mouse lemur would be associated with structural and functional deterioration of the CP, including alterations in key proteins involved in CSF homeostasis, and that these changes would be associated with age-related cognitive or motor decline. To test this hypothesis, we combined behavioral phenotyping with transcriptomic profiling and immunofluorescence analyses of CP tissue from young, adult, and aged gray mouse lemurs. We found that CP aging in this non-human primate model involves selective functional vulnerability while essential barrier and secretory functions are preserved.

## Material et methods

### Animals

All experiments were conducted in accordance with the European Directive 2010/63/EU for the protection of animals used for scientific purposes and were approved by the Cuvier Ethics Committee (committee number #68 of CNREEA [Comité national de réflexion éthique sur l’expérimentation animale]) under authorization number #13064:2018011615434702-v3. Animal welfare was ensured at every step, following the recommendations of the Weatherall report on the use of non-human primates in research. All animals were born and housed in the breeding colony of the CNRS/MNHN laboratory in Brunoy (France, agreement no. E91–114.1). They were kept under standard captive conditions: temperature at 24–26 °C, 55% relative humidity, and an artificial photoperiod mimicking seasonal changes (short-day: 10h light/14h dark and long-day: 14h light/10h dark cycles). Lighting was provided by fluorescent lamps during the day (250–350 lux) and red dim light during the night. In this study, all individuals were tested during the long-day, summer-like photoperiod. Animals were housed in groups made of 2–4 lemurs, in large cages (height = 200 cm; length = 80 cm; width = 80 cm) allowing visual, auditory and olfactory access to neighboring cages, what favors social enrichment. Cages were provided with environmental enrichment, including branches, sticks and toys. Lemurs were fed *ad libitum* with fresh fruits and vegetables and a mixture of cereals, gingerbread, yogurt, eggs, milk and water prepared daily in the laboratory. Fresh fruits and vegetables were scattered and hidden in the cages to increase enrichment. No food deprivation or nociceptive stimuli were used. Animal weights were recorded prior to euthanasia.

Animals were divided into two experimental groups. The group 1 (n = 16; 8 males, 8 females) was used for RNA sequencing analysis while the group 2 (n = 12; 6 males, 6 females) was used for histological and immunofluorescence analyses. Due to the incompatibility of CP dissection and brain slicing procedures, experiments could not be performed on the same animals. However, both groups underwent behavioral testing prior to tissue collection, including visual discrimination, spontaneous alternation, and rotarod tests. The exploratory memory task was only performed by the group 2. Behavioral testing was conducted at the Brunoy facility (France), and biological samples (CP or brain) were sent to the Université de Sherbrooke (Canada) for molecular and histological analyses. Age categories were defined as follows: young (2–4 years), adult (5–6 years), and old (≥7 years) [19].

It is important to note that the availability of aged gray mouse lemurs is inherently limited due to the restricted size of breeding colonies and the natural age distribution of the population. Consequently, unequal group sizes are common in aging studies using this non-human primate model. Sample size was determined based on an *a priori* sensitivity analysis performed with G*Power 3.1 [26] for a one-way ANOVA (three age groups), assuming a significance level of α = 0.05 and a statistical power of 80%. With the available sample sizes (RNA sequencing analysis, n = 16; histological and immunofluorescence analyses, n = 12), the study was powered to detect large effect sizes (Cohen’s f ≥ 0.87 and ≥ 1.07, respectively). These sample sizes were considered appropriate given the ethical and practical constraints associated with studies involving captive non-human primates.

### Behavioral assessments

To investigate age-related changes in motor and cognitive functions in the mouse lemur, we conducted behavioral tests targeting locomotor performance, exploratory behavior, behavioral flexibility, working memory, and visual discrimination. All tests were conducted during the animal active nocturnal phase. Data were analyzed across three age groups: young (n = 8), adult (n = 9), and old (n = 11), except when specified. Sex-specific effects were not examined in this study due to limited sample sizes within each age group.

#### Visual discrimination task.

To assess cognitive function, we employed a non-food-motivated visual discrimination paradigm previously validated in mouse lemurs [27]. The apparatus consisted of a 150 cm-high vertical cage equipped with a central jumping platform and two landing platforms: one stable (rewarded) and one unstable (non-rewarded). Animals were required to jump from the central platform to one of the two landing options, for a maximum of 30 trials per session. A correct choice allowed access to the nest box and a 2-min rest period, whereas an incorrect choice resulted in a safe fall onto a cushioned surface. In case of inaction, a gentle vibration was applied to prompt a response. Animals underwent a habituation session one day prior to the initial learning task. During the learning phase, the platforms were distinguished by highly contrasting visual cues. Each session comprised up to 30 trials conducted over one or two consecutive days and was terminated early if the animal reached the learning criterion of 8 correct choices out of 10 consecutive trials. Long-term memory was assessed one month later using the same pair of visual cues, followed the next day by a new learning session employing a different set of cues. Cognitive performance was quantified using the success rate (number of correct jumps/total trials) and the number of refusals (trials in which no jump was performed). For each experimental stage, outcomes were classified as success or failure based on predefined performance criteria.

#### Spontaneous alternation test.

Working memory and exploration strategies were assessed using a four-arm cross-maze (arms A–D) designed for mouse lemurs [28]. Animals were placed in the central area of the maze and allowed to explore freely for a 20-min session. Spontaneous alternation behavior, defined as successive entries into all four arms without repetition, was recorded through a one-way mirror to minimize experimenter interference. Only sessions with a minimum of eight arm entries were included in the analysis. The alternation percentage was calculated as follows: (number of alternations / (number of arm entries − 3)) × 100. Reduced alternation performance was interpreted as reflecting impaired working memory and/or increased anxiety-related perseverative behavior.

#### Rotarod test.

Motor coordination was assessed using an accelerating rotarod paradigm as previously described [28]. Briefly, animals were placed on a rotating rod with a progressively increasing rotation speed (from 17 to 40 rpm), and the latency to fall or voluntarily jump from the rod was recorded. Each animal completed five trials, of which the best three performances were retained for analysis. The test was terminated if the animal exhibited three consecutive passive rotations, indicative of clinging behavior. Shorter latencies were interpreted as reflecting impairments in balance, motor endurance, or adaptive motor strategies.

#### Exploration memory task.

This test was performed exclusively in group 2 (n = 12; 6 males, 6 females). Animals were allowed to explore a custom-built wooden enclosure (8 m³) consisting of four peripheral nest boxes connected to a central platform. Behavior was recorded in complete darkness using infrared cameras (1080p resolution, 30 fps) during two 20-min exploration phases. In phase 1 (baseline), animals freely explored the initial set of four nest boxes while in phase 2 (extended zone), following a 4-min rest period in the nest box, four additional nest boxes were introduced, and exploration was repeated. Video recordings were analyzed using EthoVision XT 15 software. Quantitative measures included number of platforms visited and changes in exploration patterns following environmental modification. Behavioral flexibility and working memory were inferred from changes in exploration distribution between phase 1 and phase 2.

### Histology

Following behavioral testing and euthanasia by intraperitoneal injection of pentobarbital (70 mg/kg), brains were collected and briefly rinsed in phosphate-buffered saline (**PBS**). For histological analyses, whole brains were fixed in 4% paraformaldehyde (**PFA**) for 24 h at 4 °C. Then, several washes of fresh Ethanol 70% were performed 3 x 5 min followed by incubating in ethanol 70% at 4°C until paraffin embedding. Fixed brains were sent to the Histology Platform of the Université de Sherbrooke (Canada) to be embedded in paraffin, cut in a 4µm coronal cross-section and mounted on Probe-On Plus slides (Fisher Scientific). Histological parameters of the CP were measured on brain slides stained with hematoxylin and eosin. Measurements were conducted by an experimenter blinded to age group, using two slides per brain for each age group.

### Immunofluorescence

Brain slides were first incubated two times in Citrisolv (1x) for 10 min for deparaffinization. For rehydration, brain slides were washed two times with EtOH 100% for 3 min, and then sequentially washed with EtOH 95%, EtOH 80% and water for 1 min. Slides were then incubated into boiling sodium citrate (temperature around 95°C) for 20 min and then cooled in same solution for 20 min without heat. Slides were then washed 2 times with PBS 1x. For permeabilization and blocking, slides were incubated with a blocking solution (PBS 1X + 0,1% Triton + 10% NGS) at room temperature for 2h minimum in closed humidified chamber. The primary antibody was then added to the brain sections and incubated overnight at room temperature. The next day, slides were washed 3 times for 10 min with PBS 1x and a fresh secondary antibody solution (dilution 1:1000) was added for 45 min in dark at room temperature. Slides were then washed 3 times with PBS 1x for 10 min each. A drop of Hoescht (1:500) was added on slides for 5 min incubation, then removed. A drop of slowfade Mountant (Thermo Fisher Scientific) was finally added on the center-left of the tissue with a coverslip on top. All brain slides were analyzed on the Echo Revolution microscope (Echo) with the same acquisition settings. The acquisition and analysis of images was blind for the age group.

**Table:** 

Antibody	Company	Cat n°	Dilution
NKCC1/SLC12A2	Abclonal	A11675	1:200
Transthyretin (TTR)	Abclonal	A3186	1:200
Aquaporin-1 (AQP1)	Abclonal	A15030	1:200

### RNA extraction

Total RNAs were extracted from CP tissue using a hybrid technique combining the TRIzol reagent protocol (Invitrogen, Thermo Fisher Scientific) and the Quick-RNA™ Miniprep Kit (Zymo Research) was used. CP tissues were first homogenized in 500 µL of cold TRIzol with ceramic beads using a high-speed tissue homogenizer and then 500 µL of chloroform:isoamyl alcohol 24:1 (Sigma Aldrich) were added. Samples were vortexed for 20 seconds and incubated for 2 min at room temperature. The samples were then centrifuged at 10,000g for 18 min at 4°C. The aqueous phase, that contains RNAs, was transferred in a new Eppendorf tube. An equal volume of 100% ethanol was added and the mixture was then loaded into a Zymo-Spin™ IIICG Column from the Quick-RNA™ Miniprep Kit. Further steps were conducted according to the manufacturer’s protocol. RNA concentrations and purity were determined using a Nanodrop spectrophotometer (Thermo Fisher Scientific). Only samples with 260/280 ratios above 1.8 were used for downstream applications.

### RNA-Seq library preparation

RNA-seq libraries were prepared using NEBNext® single cell/low input RNA library preparation Kit (New England Biolabs). Library preparation was initiated with 10ng of total RNA. RNA along with all the other reagents were thawed on ice. Further steps were conducted according to the manufacturer’s protocol until the final library amplification. Each final library was then purified using the MinElute PCR Purification Kit (Qiagen) and then quantified on a Qubit system (Thermo Fisher Scientific). Multiplexed libraries were pooled in approximately equimolar ratios and were then run in a 2% agarose gel. DNA between 300 and 700 base pairs was then cut and purified using the Qiagen Gel Extraction Kit (Qiagen). Library sequencing was performed at the TUCF Genomics platform (Tufts university, USA) using a HiSeq 2000 (Illumina) to generate 50 bp single end reads. The raw data have been deposited at the Gene Expression Omnibus (GEO) under the subseries entry GSE317899.

### Data analysis

Quality control of RNA-seq data was performed using FastQC (version 0.11.8) with default parameters. Trimming of the reads was achieved using TrimGalore (version 0.6.4) with default parameters except for: --illumina --max_n 5 --clip_R1 3 --three_prime_clip_R1 3. Quality of the data was also evaluated using FastQC after trimming adapters and low-quality regions. Reads were then mapped to the gray mouse lemur genome (Mmur3; GCF_000165445.2_Mmur_3.0 genome reference) with Rsubread v2.24.0. The evaluation of gene expression (CPM and TPM) was done using FeatureCounts from the SubRead package (version 2.0.0). FeatureCounts was used with default parameters except for: -M --fraction -Q 15 --fracOverlap 0.25 --largestOverlap --minOverlap 15. Exons were set as features (-t exon) and grouped by gene (-g gene_id). Statistical Analysis was performed using the edgeR package (version 3.24.3) from R (version 3.5.3) and significant changes in gene expression (5% FDR) were evaluated after filtration using the genefilter package (version 3.15). Data visualization was done using python (version 3.7.3).

## Results

### Motor and exploratory functions decrease with age

**Fig 1:**
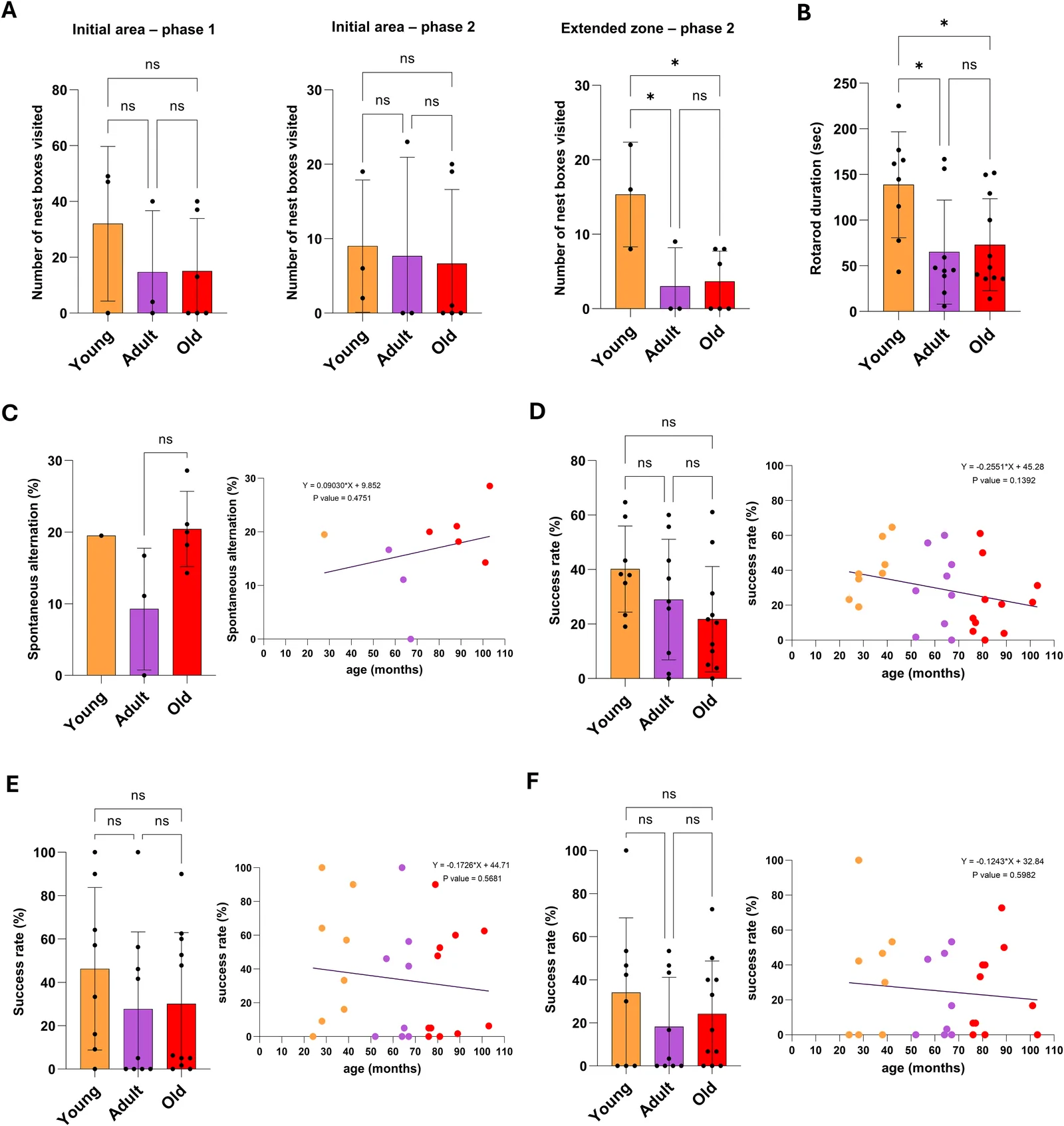
Behavioral assessments. (A) Exploration behavior task. Histograms showing the number of nest boxes visited in the initial area during the first phase (left), in the initial area during the second phase (middle) and in the extended zone during the second phase (right) (young n = 3; adult n = 3; old n = 6). (B) Motor performance. The histogram represents the average time spent on the rotarod across different age subgroups (young n = 8; adult n = 9; old n = 11). (C) Spontaneous alternation. The histogram represents the percentage of spontaneous alternation across different age subgroups (young n = 1; adult n = 3; old n = 5). On the right, individual percentages were plotted against age (in months), with the line representing the linear regression. (D-F) Visual discrimination task. Histograms showing the success rates for each mouse lemur during the first learning phase (D), the memory phase (E), and the second learning phase **(F)** of the task, grouped by age category (young n = 8; adult n = 9; old n = 11). On the right of each panel, individual scores were plotted against age (in months) for the same phases, with the line representing the linear regression. Results are indicated as mean ± SD. ns = nonsignificant, *p < 0.05 (*) from a one-way ANOVA test.

We first assessed motor and cognitive performance in young (2–4 years), adult (5–6 years), and old (≥7 years) mouse lemurs. This behavioral characterization was conducted to verify that old individuals exhibited functional signs of brain aging prior to the investigation of molecular and histological changes in the CP. Animals were subjected to behavioral tasks designed to evaluate motor coordination, exploratory behavior, working memory, and visual discrimination. We first assessed exploration behavior by measuring parameters such as the number of nest boxes visited. During the baseline phase (phase 1), no significant differences were observed among age groups in this phase (Fig 1A; left panel). In the second phase (phase 2), the originally available nest boxes were explored to a similar extent across age categories (Fig 1A; middle panel). In contrast, upon introduction of four additional nest boxes in the extended zone, young animals explored significantly more than both adult and old animals (young: 15.3 ± 7.0 boxes; adult: 3.0 ± 5.2 boxes; old: 3.7 ± 4.1 boxes) (Fig 1A; right panel), indicating an age-related decline in exploratory behavior potentially reflecting early signs of cognitive rigidity. We then assessed motor performance with the rotarod. We showed that both adult and old mouse lemurs spent significantly less time on the rotating rod compared to young animals (young: 138.8 ± 58.0 sec; adult: 65.0 ± 57.0 sec; old: 73.0 ± 50.4 sec) (Fig 1B). We also assessed spatial working memory using the spontaneous alternation task. Alternation rates did not differ significantly among age groups (young: 19.3%; adult: 9.3%; old: 20.4%) (Fig 1C). Finally, in the visual discrimination task, performance during learning (young: 40.1 ± 15.8%; adult: 29.0 ± 22.1%; old: 21.8 ± 19.3%) (Fig 1D), memory (young: 46.2 ± 37.5%; adult: 27.7 ± 35.6%; old: 30.1 ± 32.9%) (Fig 1E), and relearning phases (young: 34.0 ± 34.7%; adult: 18.1  ± 23.0%; old: 24.2 ± 24.6%) (Fig 1F) did not differ significantly across age groups. Despite considerable interindividual variability, there was a trend for visual discrimination performance to decline with age. Overall, these results suggested that motor abilities and exploratory drive decrease in aged mouse lemurs while procedural learning and long-term memory are well preserved in aging mouse lemurs.

### No significant structural changes in the aging CP

**Fig 2:**
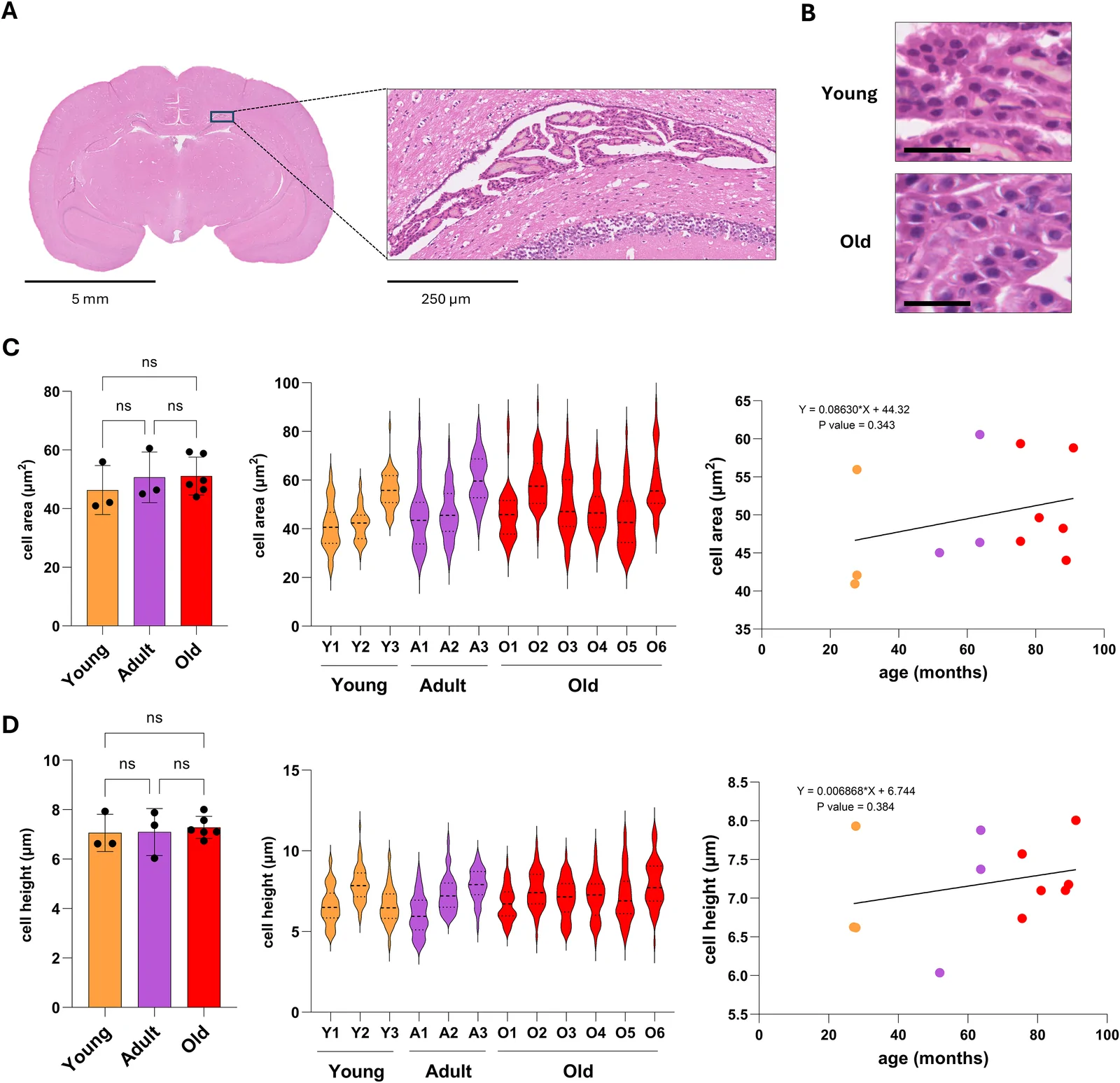
Choroid plexus morphology. (A) Representative image of the CP in the lateral ventricle on a coronal brain section from a mouse lemur, stained with hematoxylin and eosin (H&E). (B) Representative images of H&E-stained lateral ventricle CP from young (24 months) and aged (88 months) mouse lemurs. Scale bar: 25 μm. (C-D) Morphometric analysis of the lateral ventricle CP across age groups. The area **(C)** and cell height **(D)** of CP epithelial cells were quantified in young (n = 3), adult (n = 3), and old (n = 6) animals. In each panel, the left graph shows histograms of group means, with each point representing the mean of 50 measurements per individual; data are expressed as mean ± SD (ns, not significant). The middle graph displays violin plots illustrating the distribution of individual values, and the right graph depicts average cellular measurements plotted against age (in months), with the line indicating the linear regression. Young, adult and old mouse lemur CPs are respectively in orange, purple and red.

We next examined anatomical changes in the brain by analyzing hematoxylin and eosin (**H&E**)–stained brain sections from mouse lemurs of different ages (S1 Fig). The major neuroanatomical structures, including the cerebral cortex and hippocampal Cornu Ammonis (**CA**) region, showed no significant age-associated alterations in tissue organization or cytoarchitecture (S2 Fig), suggesting overall preservation of brain morphology during aging, despite a tendency toward reduced thickness in these regions. Our analysis further focused on the lateral ventricle CP, the primary site of CSF production (Fig 2A,B). To quantify potential structural alterations, we measured the height and area of CP epithelial cells (**CPECs**) across age groups. Neither cell area (young: 46.3 ± 8.3 µm²; adult: 50.7 ± 8.6 µm²; aged: 51.1 ± 6.5 µm²) (Fig 2C; S3A Fig) nor cell height (young: 7.06 ± 0.76 µm; adult: 7.09 ± 0.95 µm; old: 7.28 ± 0.44 µm) (Fig 2D; S3B Fig) differed significantly with age. Correlation analyses further confirmed the absence of a significant relationship between chronological age and these morphometric parameters (p = 0.343 for cell area; p = 0.384 for cell height) (Fig 2C,D; right panels). Overall, these findings indicate that lateral ventricle CP is well preserved during aging, with a regular epithelial architecture and consistent cellular morphology across age groups. Together with the preservation of cortical and hippocampal morphology, these results suggest that aging in the mouse lemur is not associated with widespread structural deterioration of the brain or CP. Notably, we observed no evidence of CPEC atrophy like previously reported in other animal models [29,30].

### Transcriptional remodeling of the CP during aging

**Fig 3:**
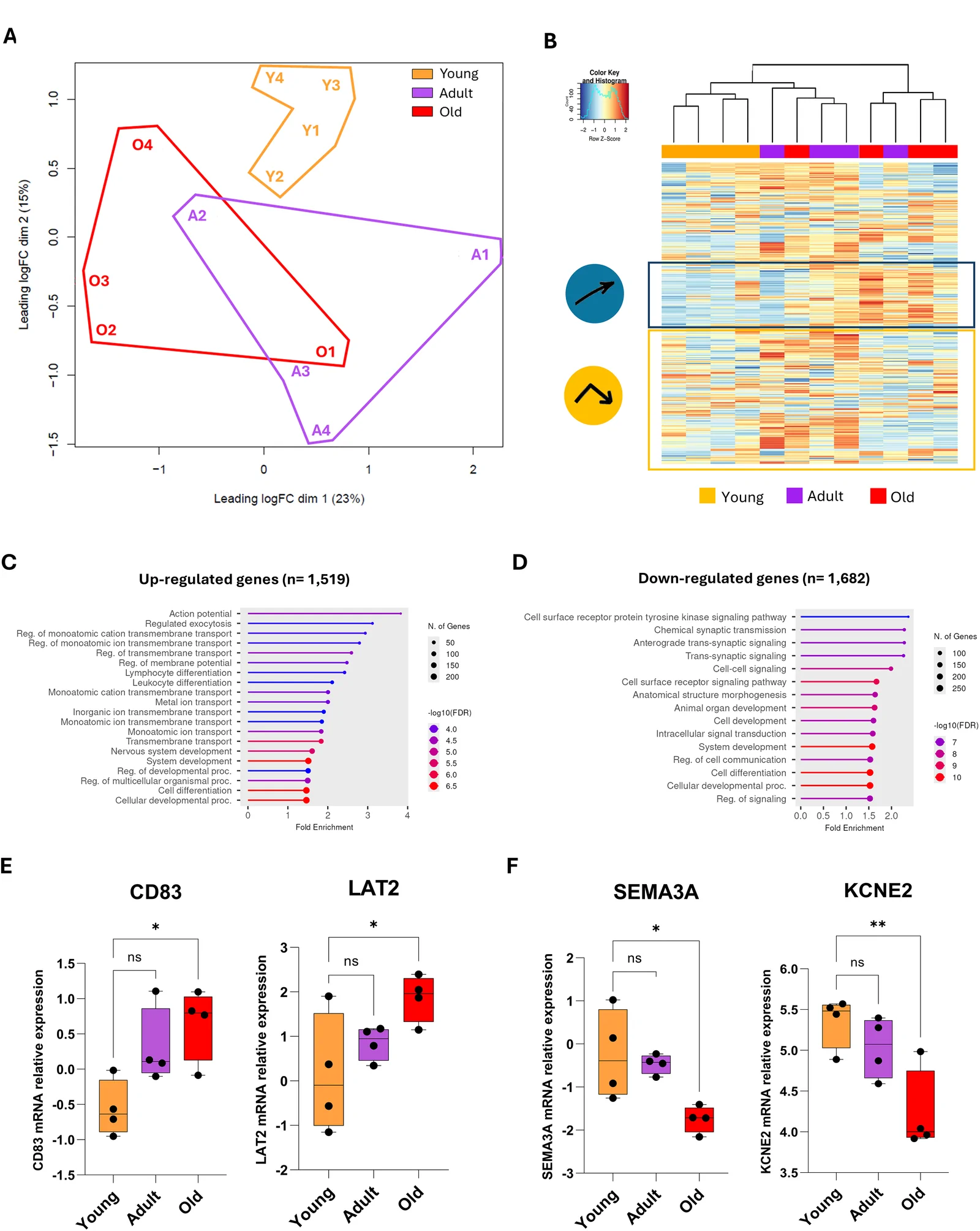
Age-related changes in the transcriptome signature of the CP. (A) Principal component analysis (PCA, explaining 38% of the variance in total) for the gene expression of young, adult and old CPs (n = 4 per age group). (B) Unsupervised hierarchical clustering of genes showing significant differential expression between young, adult and old CPs. Red and blue indicate relatively higher and lower expression, with genes independently scaled to a mean of zero. The top 500 most variable genes are represented. (C-D) Gene Ontology analysis of upregulated (n = 1,519) (**C**) and downregulated (n = 1,682) (**D**) genes using the ShinyGO tool (version 0.85). The pathway database used was GO Biological process. Parameters were set at a 0.05 FDR cut off with no redundancy in GO terms. **(E-F)** mRNA relative expression of up-regulated genes (CD83, LAT2) (**E**) and down-regulated genes (SEMA3A, KCNE2) (**F**) across age groups. Young, adult and old mouse lemurs are respectively in orange, purple and red. Results are indicated as median ± SD. ns = nonsignificant, *p < 0.05 (*) p < 0.001 (**) from a one-way ANOVA test.

We next investigated changes in mRNA expression in the aging CP. We microdissected the lateral ventricle CP from young, adult and old mouse lemurs (n = 4 for each age group), extracted total RNAs from the tissue and then performed a transcriptomic analysis. First, the principal component analysis (**PCA**) showed that young samples (Y1-Y4) clustered distinctly and occupied a relatively compact region of the plot, indicating homogeneous transcriptional profiles (Fig 3A). Adult samples (A1-A4) showed greater dispersion, suggesting increased inter-individual variability with age. Old samples (O1-O4) were clearly separated from young samples along the first principal component, responsible for 23% of the variance, and partially overlap with the adult group, consistent with progressive age-related transcriptional remodeling of the CP. Overall, the separation of age groups highlighted age-dependent changes in CP gene expression, with increasing heterogeneity in adult and old CPs.

We then analyzed each individual gene and their differential expression between each age group. We selected the 500 most variable genes and represented them in a heatmap (Fig 2B). Unsupervised hierarchical clustering of samples (dendrogram at the top) for these 500 genes revealed an age-dependent organization of transcriptional profiles, with young samples clustering separately from adult and old samples (Fig 2B). Two major gene clusters were found (boxed regions), corresponding to distinct age-associated expression patterns. One cluster was characterized by increased expression in old samples compared to young samples. In this cluster, there was an overrepresentation of immunoglobulins (**Ig**) among age-related variable genes (S1 Table). Another cluster showed a specific increased expression in adult CP. These different patterns suggest a coordinated transcriptional remodeling of the CP during aging.

The analysis of genes exhibiting substantial expression changes (≥40% differential expression) between young and old CPs identified 1,519 upregulated genes and 1,682 downregulated genes (Fig 3C). To identify the biological pathways associated with these genes, we performed gene ontology (**GO**) analysis using ShinyGO, an in-depth gene set enrichment tool [31]. Upregulated genes (n = 1,519) were predominantly enriched in biological processes related to ion and electrical transport, as well as immune-related functions (Fig 3C). In contrast, downregulated genes (n = 1,682) were mainly associated with cell signaling and intercellular communication pathways (Fig 3D). Among the DEGs, several candidates were closely related to CP functions. CD83 was one of the most significantly upregulated genes and showed a strong positive correlation with age (Fig 3E; S2 Fig). CD83 is an immune regulatory molecule expressed by activated antigen-presenting cells and its upregulation is associated with interferon-driven immune responses in CPECs [32,33], indicating a shift of the CP toward a more immune-alert or pro-inflammatory state during aging. LAT2, a neutral amino acid transporter expressed in CPECs and involved in CSF composition [34], was also upregulated (Fig 3E; S2 Fig), potentially affecting CSF nutrient balance and brain metabolic resilience during aging. Among downregulated genes, the expression of SEMA3A, normally secreted into the CSF to regulate neuronal plasticity, synaptic organization, and immune cell recruitment [35–37], was reduced (Fig 3F; S2 Fig). Finally, KCNE2, a β-subunit of voltage-gated potassium channels and one of the most enriched ion channel regulators in the CP, was downregulated (Fig 3F; S2 Fig). Loss of KCNE2 has been shown to markedly impair CSF production [38], suggesting compromised CSF homeostasis with aging.

Together, these results demonstrate that aging is associated with a progressive and coordinated transcriptional remodeling of the CP. This remodeling is characterized by increased inter-individual heterogeneity and altered expression of key CP regulators involved in immunity, nutrient transport, neuronal signaling, and CSF production.

### AQP1 protein expression declines in the aged CP

**Fig 4:**
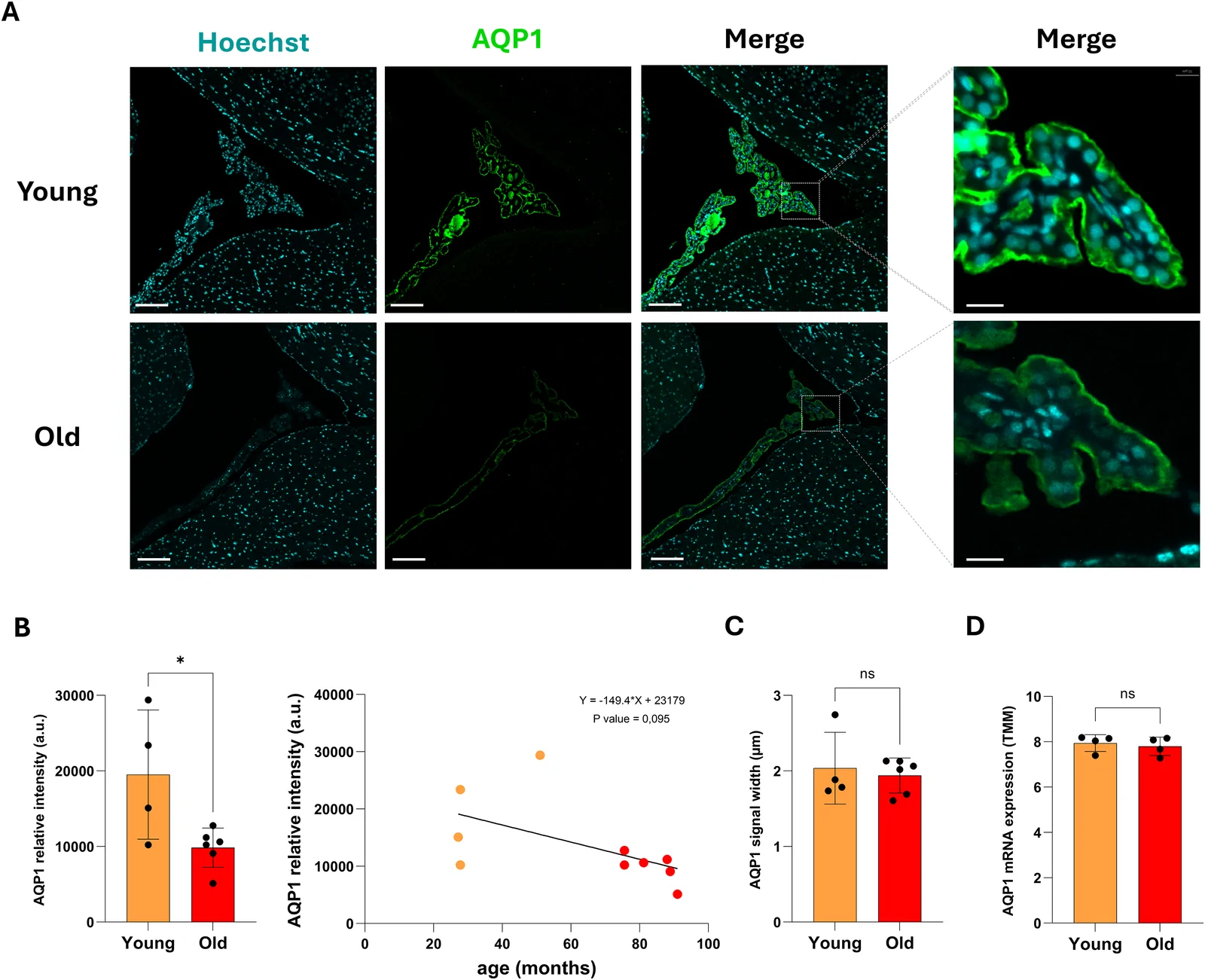
Analysis of AQP1 expression in the aging CP. (A) AQP1 immunofluorescence on coronal cuts of young and old brains. Images illustrate the representative staining in the CP area with the AQP1 antibody (green) and Hoescht (blue). Scale bar: 100 μm on the left panel and 10 μm on the right panel (higher magnification). (B) The left graph shows the quantitative analysis of AQP1 signal intensity (young n = 4; old n = 6) with each point representing the mean of 20 to 30 measurements per individual; data are expressed as mean ± SD. *p < 0.05 (*) from an unpaired t-test. The right graph depicts signal intensity measurements plotted against age (in months), with the line indicating the linear regression. (C) Quantitative analysis of AQP1 signal width (young n = 4; old n = 6) with each point representing the mean of at least 50 measurements per individual; data are expressed as mean ± SD. ns = nonsignificant from an unpaired t-test. **(D)** mRNA levels of AQP1 measured by RNA-seq analysis (n = 4 per age group). Results are indicated as mean ± SD. ns = nonsignificant from an unpaired t-test.

We next investigated the age-related changes in CP main functions. We first examined AQP1 expression as it plays a key role in the CP, where it is the principal water channel responsible for CSF production and regulation [39]. Indeed, genetic deletion or pharmacological inhibition of AQP1 leads to reduced CSF production [40]. We performed immunofluorescence on young and old brain tissue. AQP-1 immunostaining on coronal cuts of young brains showed a specific signal localized to the CP (Fig 4A). In contrast, surrounding tissue displays weaker or minimal AQP1 signal. Higher-magnification images highlight that AQP1 was concentrated along the apical (CSF-facing) membrane of CPECs, consistent with its role as a transmembrane water channel (Fig 4A, top right panels). We observed a strong and significant decrease in AQP1 signal in old CPs with low intra-group variability (young: 19,518 ± 8,574 a.u.; old: 9,829 ± 2,603 a.u.) (Fig 4B; S2A Fig). The overall tissue architecture and cellularity appeared preserved as indicated by Hoechst staining. Interestingly, we found that the width of AQP1 signal remained unchanged between the two age groups (young: 2.04 ± 0.47 µm; old: 1.94 ± 0.23 µm) (Fig 4C; S2B Fig), implying that the observed differences are specific to AQP1 expression rather than global tissue loss or reduced cellularity. We finally investigated whether the observed reduction in AQP1 signal reflected transcriptional or post-transcriptional regulation. RNA-seq analysis revealed comparable AQP1 mRNA levels in young and aged CPs (young: 7.94 ± 0.37 TMM; old: 7.80 ± 0.40 TMM) (Fig 4D), indicating that AQP1 is regulated predominantly at the post-transcriptional level. Together, these results demonstrated a marked age-associated reduction of AQP1 protein expression in the CP, potentially contributing to altered CSF production in the aged brain.

### NKCC1 expression is maintained during CP aging

**Fig 5:**
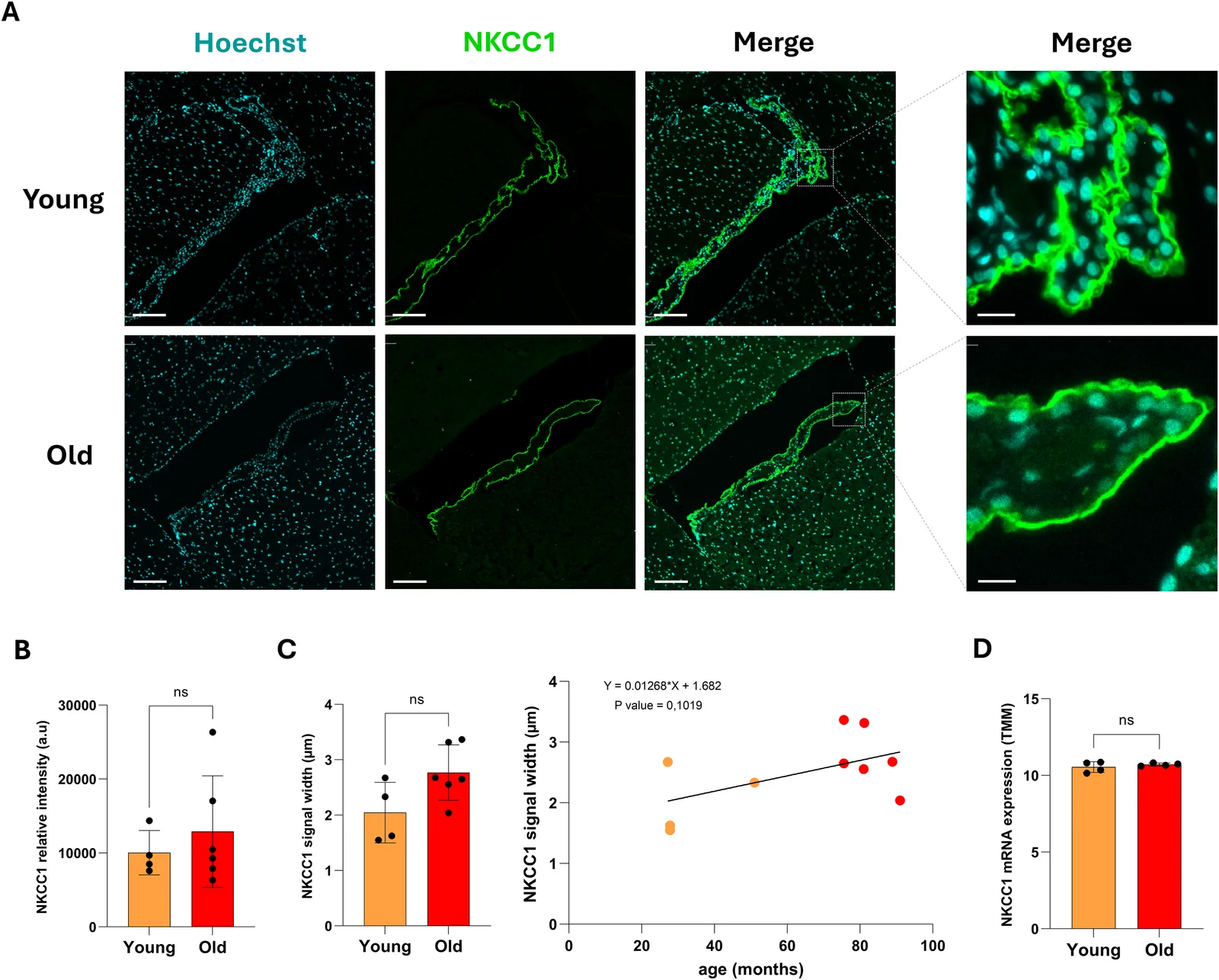
Analysis of NKCC1 expression in the aging CP. (A) NKCC1 immunofluorescence on coronal cuts of young and old brains. Images illustrate the representative staining in the CP area with the NKCC1 antibody (green) and Hoescht (blue). Scale bar: 100 μm on the left panel and 10 μm on the right panel (higher magnification). (B) Quantitative analysis of NKCC1 signal intensity (young n = 4; old n = 6) with each point representing the mean of at least 400 measurements per individual; data are expressed as mean ± SD. ns = nonsignificant from an unpaired t-test. (C) The left graph shows the quantitative analysis of NKCC1 signal width (young n = 4; old n = 6) with each point representing the mean of at least 50 measurements per individual; data are expressed as mean ± SD. ns = nonsignificant from an unpaired t-test. The right graph depicts signal width measurements plotted against age (in months), with the line indicating the linear regression. **(D)** mRNA levels of NKCC1 measured by RNA-seq analysis (n = 4 per age group). Results are indicated as mean ± SD. ns = nonsignificant from an unpaired t-test.

AQP1 works in concert with transporters such as Na ⁺ /K ⁺ -ATPase, carbonic anhydrases, and anion exchangers to establish the osmotic gradients that drive water movement. We next examined the expression of NKCC1 (also known as SLC12A2) as it is a key ion transporter in the CP and plays a central role in CSF production and ionic homeostasis [41]. NKCC1 helps maintain proper Na ⁺ , K ⁺ , and Cl_−_ concentrations in the CSF, and this tight control of CSF electrolyte composition is critical for neuronal excitability and brain homeostasis [42]. Pharmacological inhibition or genetic manipulation of NKCC1 reduces CSF production rates, demonstrating its functional importance. We performed immunofluorescence analyses on brain tissue from young and aged mouse lemurs. In young brains, NKCC1 immunostaining on coronal sections revealed a strong and specific signal localized to the CP (Fig 5A), whereas surrounding tissues exhibited little to no signal. Like AQP1, NKCC1 signal was enriched along the apical membrane of CPECs, consistent with its function as an ion transporter (Fig 5A, top right panels). In aged CP, NKCC1 signal was not significantly altered, although a trend toward increased expression was observed (young: 10,035 ± 3,008 a.u.; old: 12,877 ± 7,551 a.u.) (Fig 5B; S2C Fig). Similarly, the width of the NKCC1 signal remained comparable between age groups (young: 2.04 ± 0.55 µm; old: 2.77 ± 0.50 µm) (Fig 5C; S2D Fig), as did NKCC1 mRNA levels (young: 10.54 ± 0.35 TMM; old: 10.70 ± 0.10 TMM) (Fig 5D). Together, these results indicated that, unlike AQP1, NKCC1 expression is largely preserved with aging in the CP. This suggested that age-related alterations in CSF production are unlikely to result from changes in NKCC1 abundance and instead may arise from post-transcriptional regulation of water transport.

### Stable TTR expression but increased cellular heterogeneity in the aging CP

Finally, we analyzed the expression of TTR which is one of the most abundant proteins secreted by the CP into the CSF. In rodents, CP TTR expression often decreases with age, correlating with cognitive decline while in humans, CSF TTR levels tend to increase during healthy aging [43,44]. We performed immunofluorescence analyses on brain tissue from young and aged mouse lemurs.

**Fig 6:**
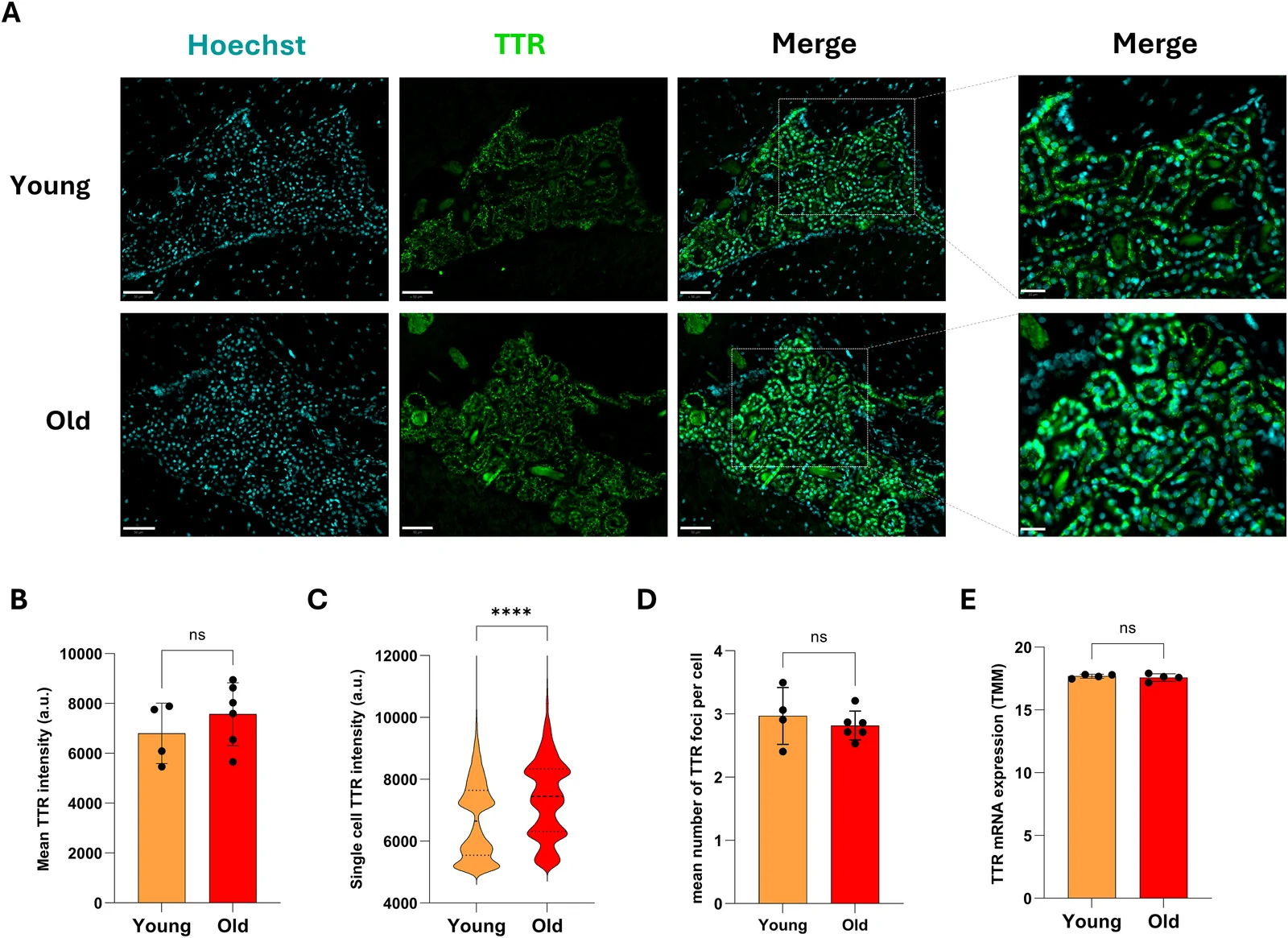
Analysis of TTR expression in the aging CP. (A) TTR immunofluorescence on coronal cuts of young and old brains. Images illustrate the representative staining in the CP area with the TTR antibody (green) and Hoescht (blue). Scale bar: 50 μm on the left panel and 10 μm on the right panel (higher magnification). (B) Quantitative analysis of TTR signal intensity (young n = 4; old n = 6) with each point representing the mean of 600 measurements per individual; data are expressed as mean ± SD. ns = nonsignificant from an unpaired t-test. (C) Violin plot of single cell measurement of TTR signal with a minimum of 200 measurements per CP, representing a total of 6017 measurements for young CPs (n = 4) and 9658 measurements for old CPs (n = 6). p < 0.0001 (****) from an unpaired t-test with Welch’s correction. (D) Graph depicting the number of TTR foci per CP epithelial cells in young and old CPs (young n = 4; old n = 6). Results are indicated as mean ± SD. ns = nonsignificant from an unpaired t-test. **(E)** mRNA levels of TTR measured by RNA-seq analysis (n = 4 per age group). Results are indicated as mean ± SD. ns = nonsignificant from an unpaired t-test.

Overall TTR expression remained high during aging, with no significant change in mean signal intensity between age groups (young: 6,799 ± 1,210 a.u.; old: 7,567 ± 1,261 a.u.) (Fig 6A,B). In contrast, single-cell analysis (50 measurements per individual) revealed a significant increase in TTR signal intensity in CPECs of aged individuals (Fig 6C), highlighting increased cellular heterogeneity not detected by mean-level analysis. As TTR immunostaining displayed a foci-like signal inside CPECs, we next quantified the number of TTR foci per cell but did not find any differences between the two groups (young: 2.97 ± 0.45 foci, old: 2.82 ± 0.23 foci) (Fig 6D). Similarly, transcriptomic analyses did not show any significant variation in TTR gene expression (young: 17.69 ± 0.16 TMM; old: 17.57 ± 0.30 TMM) (Fig 6E). Together, these results indicated that TTR expression in the CP do not show any significant change overall. However, single-cell analysis revealed increased variability in TTR signal among CPECs in old individuals, indicating enhanced cellular heterogeneity.

### Correlation of CP functional markers with age-related motor decline

**Fig 7:**
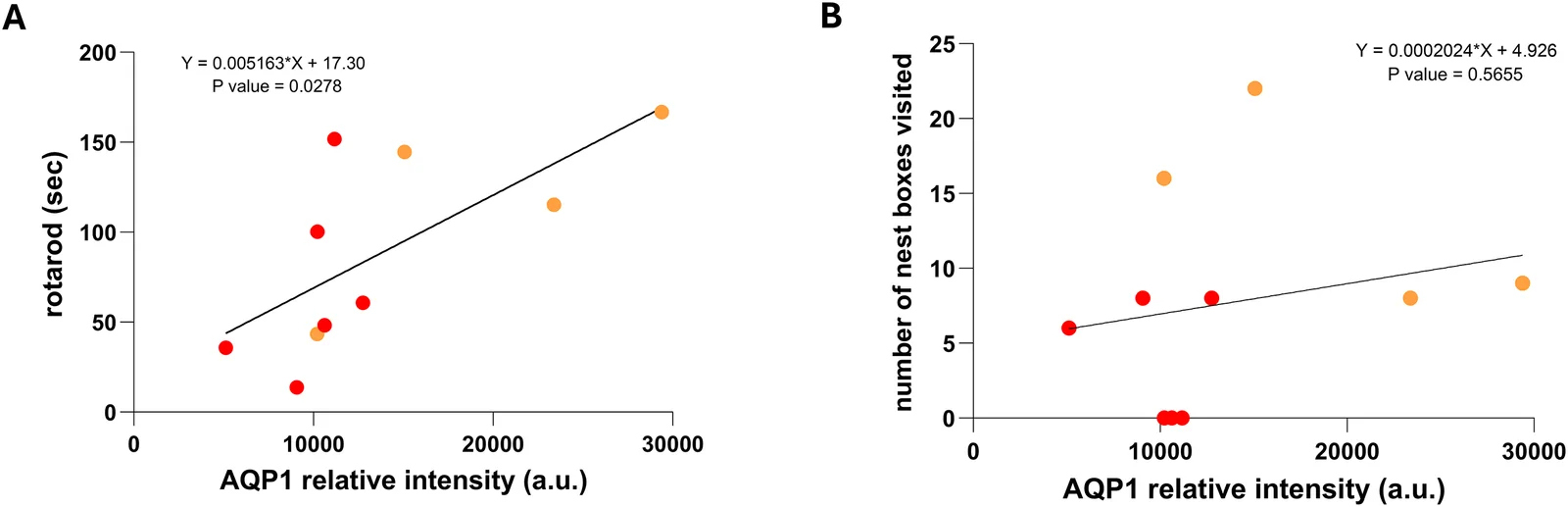
Correlation of AQP1 expression with behavior functions. (A-B) Graphs depicting the motor performance with the average time spent on the rotarod (Fig 1B) (A) and the exploration behavior with the number of nest boxes visited in the extended zone (Fig 1A; right panel) **(B)** plotted against AQP1 signal intensity (Fig 4B), with the line indicating the linear regression. Young (n = 4) and old (n = 6) mouse lemurs are respectively orange and red.

We observed transcriptional and functional alterations in the mouse lemur CP with aging, but it remained unclear whether these changes were linked to cognitive performance. Behavioral assessments revealed that only motor abilities (rotarod) and exploratory drive were reduced in aged animals (Fig 1). Functionally, aged CPs showed a notable decrease in AQP1 protein expression (Fig 4) and increased cellular variability in TTR signal among CPECs (Fig 6). We examined potential correlations between these CP markers and behavioral changes. While no significant correlation was detected for TTR (data not shown), AQP1 expression in the CP positively correlated with rotarod performance during aging (Fig 7A) but showed no association with exploratory behavior (Fig 7B). These results suggest that CP water transport capacity may contribute to age-related motor decline.

## Discussion

Aging of the brain is a multifaceted process involving behavioral, molecular, and structural changes that vary markedly across species. The present study provides the first multimodal characterization of age-related changes in the CP of the gray mouse lemur (*Microcebus murinus*), an emerging non-human primate model. By combining behavioral testing with transcriptomic, morphological, and molecular analyses, we uncover the preservation of CP core functions alongside selective vulnerabilities.

### Behavioral aging: dissociation between motor decline and cognitive preservation

At the behavioral level, aging in mouse lemurs was associated with a clear decline in motor performance, as evidenced by reduced latency on the rotarod test in adult and aging individuals (Fig 1B). This finding is consistent with previous work in mouse lemurs showing that impairments in balance and coordination can emerge relatively early, with deficits on the Rotarod reported sometimes from mid-adulthood [28,45,46]. Unlike terrestrial laboratory rodents, mouse lemurs are small, primarily arboreal primates whose natural locomotion involves navigating narrow and often unstable branches, climbing, and jumping between supports. These behaviors place particular demands on hindlimb strength, grip, proprioception, and rapid postural adjustments. Consequently, age-related changes in any of these functions may have a pronounced impact on rotarod performance, which requires sustained balance and coordinated hindlimb control on a rotating and progressively challenging surface. The observed decline may therefore reflect a combination of neurobiological and peripheral factors, including impaired proprioception, reduced muscle strength, sarcopenia, and slower postural reflexes [47]. In particular, age-related reductions in lower-limb and foot grip strength reported in mouse lemurs may be especially relevant because strong hindlimb and foot control are essential for maintaining stability during arboreal locomotion. Importantly, reduced performance on the Rotarod should not necessarily be interpreted as a generalized loss of motor capacity. Aging mouse lemurs have been shown to favor energetically conservative and less risky locomotor behaviors, avoiding unstable supports and reducing jumping behavior [45,48]. Such behavioral adjustments may compensate for declining strength and coordination by reducing the biomechanical and energetic demands of locomotion and minimizing the risk of falls. These species-specific features should be considered when interpreting mouse lemur motor performance and when extrapolating findings to humans. Although the underlying processes of age-related muscle weakness, proprioceptive decline, and impaired postural control may be conserved across primates, the magnitude and functional consequences of these changes are likely influenced by the distinct locomotor demands of an arboreal species.

In contrast to motor performance, other cognitive functions were relatively preserved with age in our study as reported by no significant age-related differences in spontaneous alternation, visual discrimination learning, or procedural memory (Fig 1). Although these findings must be interpreted cautiously given the limited sample size and high interindividual variability, they are broadly consistent with previous studies in non-human primates and humans [18,49]. In particular, procedural memory, that largely dependent on cerebellar circuits, has been shown to remain stable across aging in both species [50]. The absence of age-related decline in spontaneous alternation also suggests that short-term spatial working memory may be relatively resistant to aging in mouse lemurs. This resilience may be linked to preserved hippocampal volume, as recent MRI studies have shown internal hippocampal reorganization rather than global atrophy, with substantial interindividual variability [51]. Only a subset of aged individuals exhibit pronounced hippocampal degeneration, which may explain why cognitive impairments are difficult to detect at the group level. Including older animals in the cohort could have revealed more pronounced age-related changes in cognitive decline.

However, aging did affect exploratory behavior and behavioral flexibility. Older mouse lemurs had a reduced preference for novelty, tending to revisit familiar nest boxes rather than explore newly introduced ones (Fig 1A). This pattern aligns with earlier observations in primates, including humans, where sensitivity to novelty and exploratory drive decline with age [52]. This shift may be driven by increased anxiety toward environmental change. Reduced exploratory flexibility is potentially consequential, as exploration and environmental mapping are critical for foraging and predator avoidance. Accordingly, subtle changes in motivation and flexibility – despite intact memory – may capture important aspects of cognitive aging.

### Transcriptomic signatures of CP aging: immune activation and functional reorganization

At the molecular level, transcriptomic analyses of the lateral ventricle CP revealed a clear age-related signature. PCA analysis showed a marked separation between young and old individuals, indicating that aging strongly influences CP gene expression. Notably, adult and aged groups exhibited greater heterogeneity, highlighting variability in aging trajectories within this species. An important finding was the overrepresentation of immunoglobulins (Ig) among the most variable genes (Fig 3B), suggesting that immune-related processes are particularly sensitive to aging in the CP. This observation is consistent with growing evidence that aging tissues accumulate immunoglobulins, especially IgG, in association with cellular senescence and chronic inflammation [53]. This finding supports a model in which CP aging is closely linked to immune dysregulation, potentially influencing broader brain aging processes.

Differential expression analysis revealed considerable heterogeneity, contrasting with the more homogeneous transcriptomic profiles typically observed in rodent models. This likely reflects the high genetic diversity of mouse lemurs compared with inbred rodent strains. Nevertheless, among the genes differentially expressed during CP aging, we identified key CP regulators involved in immunity, nutrient transport, neuronal signaling, and CSF production (Fig 3E,F) suggesting that aging shifts the CP toward an immune-alert state while potentially compromising its capacity to support optimal CSF homeostasis and brain communication.

### Functional reorganization: preservation of core CP functions

Functional enrichment analyses revealed a clear dichotomy in age-related transcriptional changes. Genes upregulated with age were primarily associated with ion transport and membrane potential regulation, important functions central to CSF production. This enrichment suggests that aging CP may engage compensatory mechanisms to preserve essential physiological roles, particularly ionic homeostasis and fluid production. Conversely, genes downregulated with age were mainly involved in cellular signaling, intercellular communication, and synaptic-related pathways. The CP is not merely a secretory organ but also an important signaling hub, releasing hormones, growth factors, and signaling molecules into the CSF that influence brain development, plasticity, and homeostasis. Reduced expression of communication-related genes therefore suggests a gradual decline in CP–brain signaling capacity. This shift may represent a trade-off in aging, whereby maintenance of essential barrier and secretory functions is prioritized at the expense of regulatory and signaling roles. Such a reallocation of functional resources could have important consequences. Altered CSF-mediated signaling may contribute to neuroinflammation, reduced synaptic plasticity, and vulnerability to cognitive decline. This model aligns with emerging views of brain aging as a process characterized by dysregulated communication and homeostasis rather than by uniform structural degeneration.

### Molecular specificity of CP aging in the mouse lemur: a primate-like profile

At the molecular level, detailed analysis of key CP transporters and secreted proteins revealed a pattern distinct from that described in rodents and more closely aligned with humans. Morphologically, CPECs in mouse lemurs exhibited remarkable stability with age, with no major changes in cell height or area. This contrasts with mice, where aging is associated with epithelial flattening [54]. The structural resilience observed in mouse lemurs may relate to their longer lifespan and primate physiology.

Functionally, major transport systems involved in CSF production were largely preserved. NKCC1 expression remained stable at both transcript and protein levels, suggesting maintenance of ionic transport capacity (Fig 5). Although NKCC1 function can be modulated by phosphorylation and ionic gradients, its apparent resistance to aging-related modulation is consistent with reports showing that NKCC1 remains robust even under inflammatory conditions [55]. In contrast, AQP1 protein levels decreased with age without changes in mRNA expression, indicating post-transcriptional regulation (Fig 4). Notably, membrane localization remained intact, suggesting compensatory mechanisms that preserve water permeability despite reduced protein abundance. This contrasts with mice, where aging induces both transcriptional downregulation and impaired membrane targeting of AQP1, contributing to diminished CSF production [15]. In our study, AQP1 reduction correlated with age-related motor decline (Fig 7), implying that CP water transport capacity may influence motor function, which depend on coordinated neural networks sensitive to subtle homeostatic disturbances. In humans and non-human primates, AQP1 decline is often accompanied by compensatory AQP4 expression to maintain transcellular water transport [56]. The mouse lemur may employ a primate-like strategy, relying on post-transcriptional regulation and compensation rather than transcriptional collapse.

Transthyretin, the main protein secreted by the CP, also exhibited a primate-like aging profile. While overall TTR levels remained high, single-cell analyses revealed increased heterogeneity with age, suggesting dysregulation of proteostasis rather than reduced production (Fig 6). In rodents and large mammals such as sheep, aging is associated with reduced TTR secretion [57–59], whereas in humans, CSF TTR levels often increase during healthy aging but decline sharply in Alzheimer’s disease [60,61]. The mouse lemur thus appears to recapitulate key aspects of human CP aging, characterized by preserved production alongside altered regulation.

### Limitations

Several limitations should be acknowledged. Sample sizes were modest, particularly for transcriptomic analyses, which constrained statistical power and limited the ability to examine sex-specific differences. High interindividual variability, a defining feature of mouse lemurs, further complicates biomarker identification. The cross-sectional design precludes analysis of individual aging trajectories, and the use of whole CP tissue prevents cell-type–specific resolution of transcriptomic changes. An additional limitation concerns the assessment of CPEC morphology. Cell height was evaluated using conventional 4-µm-thick histological sections, which may not provide sufficient resolution to reliably detect subtle differences on the scale of a few micrometers. Therefore, the absence of significant age-related differences in cell height should be interpreted with caution and does not necessarily exclude subtle morphological changes. Higher-resolution approaches, such as semi-thin sections or electron microscopy, would provide more precise measurements of CP epithelial cell morphology and may reveal age-related changes, like previously reported in other animal models, that were not detectable using the current histological approach. Finally, the absence of CSF samples limits functional interpretation.

## Conclusions and perspectives

Taken together, our findings indicate that CP aging in the gray mouse lemur is not characterized by a generalized loss of structural integrity or core barrier and secretory functions. Rather, its aging involves selective molecular and functional vulnerability, including transcriptional remodeling, immune activation, and reduced AQP1 protein expression. Importantly, AQP1 expression was positively associated with motor performance, suggesting that altered CP water transport may be linked to age-related motor decline. Therefore, our hypothesis that CP aging would be associated with functional deterioration is only partially supported: aging remodels CP function and molecular state, but substantial preservation of essential CP functions is maintained. These findings support a model in which the aging mouse lemur CP undergoes functional reorganization and selective vulnerability rather than uniform degeneration. Therefore, the mouse lemur exhibits aging features closer to humans than to rodents, reinforcing its value for translational research. Future studies integrating single-cell transcriptomics, CSF analyses, and human-relevant models such as CP organoids will be critical to further dissect CP aging mechanisms and identify therapeutic targets to preserve human brain homeostasis during aging.

## Supporting information

S1 Table Top 500 most variable genes across samples. Values are counts per million (cpm).
(XLSX)

S1 Fig Mouse lemur brain anatomy across aging. (A) Representative coronal brain section stained with hematoxylin and eosin (H&E), illustrating the comparison between young and aged animals. The dashed line indicates the midline used to juxtapose the two hemispheres. Scale bar = 5 mm. **(B)** Representative H&E-stained coronal brain sections from female (♀) and male (♂) animals at young (27.2–27.8 months), adult (63.7–75.6 months), and aged (81.2–88.0 months) stages. Scale bar = 1 cm.
(TIF)

S2 Fig Histological assessment and quantitative analysis of cortical and hippocampal morphology during aging. (A) Representative coronal brain sections from young and aged animals stained with hematoxylin and eosin (H&E). Higher-magnification views of the cerebral cortex and hippocampal Cornu Ammonis (CA) regions are shown for the areas indicated by boxed regions. Scale bars = 5 mm for whole-brain sections and 100 μm for magnified views. **(B)** Representative coronal section illustrating measurements (yellow arrows) of cortical thickness (left panel) and hippocampal CA layer thickness (right panel). Scale bar = 5 mm. Below the section, quantitative analyses comparing cortex (left) and hippocampal CA (right) regions for young, adult, and aged animals are presented as mean ± SEM. ns = nonsignificant from a one-way ANOVA test.
(TIF)

S3 Fig Distribution of CP epithelial cell morphometric parameters across age groups. Histograms illustrate the distribution of individual measurements of CP epithelial cell (CPEC) area **(A)** and cell height **(B)** in young (left, orange), adult (middle, purple), and old (right, red) mouse lemurs. Each histogram represents pooled measurements obtained from the lateral ventricle CP, with values derived from multiple cells per individual.
(TIF)

S4 Fig Representative genes up- and downregulated in the aging CP. Graphs depicting average gene expression of up-regulated genes (CD83, LAT2; top panel) and down-regulated genes (SEMA3A, KCNE2; bottom panel) plotted against age (in months), with the line indicating the linear regression. Young, adult and old mouse lemurs are respectively in orange, purple and red.
(TIF)

S5 Fig Quantification of AQP1, NKCC1, and TTR signals obtained from immunofluoresence in the aging CP. (A-B) Violin plots show AQP1 signal intensity **(A)** and width **(B)** measurements in CP epithelial cells from individual young (n = 4; Y1-Y4; orange) and old (n = 6; O1-O6; red) CPs. Each violin represents a minimum of 50 pooled measurements from one individual. **(C-D)** Violin plots show NKCC1 signal intensity **(C)** and width **(D)** measurements in CP epithelial cells from individual young (n = 4; Y1-Y4; orange) and old (n = 6; O1-O6; red) CPs. Each violin represents a minimum of 50 pooled measurements from one individual. **(E-F)** Violin plots show TTR signal intensity **(E)** and the number of TTR foci per cell **(F)** in CP epithelial cells from individual young (n = 4; Y1-Y4; orange) and old (n = 6; O1-O6; red) CPs. Each violin represents a minimum of 50 pooled measurements from one individual.
(TIF)

## References

[1] Iliff JJ, Wang M, Liao Y, Plogg BA, Peng W, Gundersen GA, et al. A paravascular pathway facilitates CSF flow through the brain parenchyma and the clearance of interstitial solutes, including amyloid β. Sci Transl Med. 2012;4(147):147ra111. doi: 10.1126/scitranslmed.3003748 22896675 PMC3551275

[2] Mestre H, Du T, Sweeney AM, Liu G, Samson AJ, Peng W, et al. Cerebrospinal fluid influx drives acute ischemic tissue swelling. Science. 2020;367(6483):eaax7171. doi: 10.1126/science.aax7171 32001524 PMC7375109

[3] Liddelow A. Development of the choroid plexus and blood-CSF barrier. Frontiers in Neuroscience. 2015. 10.3389/fnins.2015.00032 PMC4347429 25784848

[4] Ya’el C. Choroid Plexus Pathophysiology. Annu Rev Pathol. 2025. 10.1146/annurev-pathmechdis-051222-114051 PMC11884907 39383438

[5] Saunders NR, Dziegielewska KM, Fame RM, Lehtinen MK, Liddelow SA. The choroid plexus: a missing link in our understanding of brain development and function. Physiol Rev. 2023;103(1):919–56. doi: 10.1152/physrev.00060.2021 36173801 PMC9678431

[6] Fame RM, Lehtinen MK. Emergence and Developmental Roles of the Cerebrospinal Fluid System. Dev Cell. 2020;52(3):261–75. doi: 10.1016/j.devcel.2020.01.027 32049038 PMC12234156

[7] Serot JM. Morphological alterations of the choroid plexus in late-onset Alzheimer’s disease. Acta Neuropathologica. 2000. 10.1007/pl00007412 10672315

[8] Gong Z. Age-related differences in the choroid plexus structural integrity are associated with changes in cognition. medRxiv. 2025. 10.1186/s12987-025-00749-3 PMC12829252 41413527

[9] Silva-Vargas V, Maldonado-Soto AR, Mizrak D, Codega P, Doetsch F. Age-Dependent Niche Signals from the Choroid Plexus Regulate Adult Neural Stem Cells. Cell Stem Cell. 2016;19(5):643–52. doi: 10.1016/j.stem.2016.06.013 27452173

[10] Brown DP. Molecular mechanisms of cerebrospinal fluid production. Neuroscience. 2004. 10.1016/j.neuroscience.2004.07.003 PMC1890044 15561411

[11] Damkier HH, Brown PD, Praetorius J. Cerebrospinal fluid secretion by the choroid plexus. Physiol Rev. 2013;93(4):1847–92. doi: 10.1152/physrev.00004.2013 24137023

[12] Viggars AP, Wharton SB, Simpson JE, Matthews FE, Brayne C, Savva GM, et al. Alterations in the blood brain barrier in ageing cerebral cortex in relationship to Alzheimer-type pathology: a study in the MRC-CFAS population neuropathology cohort. Neurosci Lett. 2011;505(1):25–30. doi: 10.1016/j.neulet.2011.09.049 21970975

[13] Marques F, Sousa JC, Coppola G, Geschwind DH, Sousa N, Palha JA, et al. The choroid plexus response to a repeated peripheral inflammatory stimulus. BMC Neurosci. 2009;10:135. doi: 10.1186/1471-2202-10-135 19922669 PMC2784788

[14] Baruch K, Deczkowska A, David E, Castellano JM, Miller O, Kertser A, et al. Aging. Aging-induced type I interferon response at the choroid plexus negatively affects brain function. Science. 2014;346(6205):89–93. doi: 10.1126/science.1252945 25147279 PMC4869326

[15] Sadanandan J. Aging alters the expression of trophic factors and tight junction proteins in the mouse choroid plexus. Fluids and Barriers of the CNS. 2024. 10.1186/s12987-024-00574-0 PMC11438291 39334352

[16] Marques F. Transcriptome signature of the adult mouse choroid plexus. Fluids and barriers of the CNS. 2011. 10.1186/2045-8118-8-10 PMC3042978 21349147

[17] Eisma JJ, McKnight CD, Hett K, Elenberger J, Song AK, Stark AJ, et al. Choroid plexus perfusion and bulk cerebrospinal fluid flow across the adult lifespan. J Cereb Blood Flow Metab. 2022;43(2):269–80. doi: 10.1177/0271678x221129101 36200473 PMC9903224

[18] Trouche SG, Maurice T, Rouland S, Verdier J-M, Mestre-Francés N. The three-panel runway maze adapted to Microcebus murinus reveals age-related differences in memory and perseverance performances. Neurobiol Learn Mem. 2010;94(1):100–6. doi: 10.1016/j.nlm.2010.04.006 20403446 PMC2881169

[19] Languille S, Blanc S, Blin O, Canale CI, Dal-Pan A, Devau G, et al. The grey mouse lemur: a non-human primate model for ageing studies. Ageing Res Rev. 2012;11(1):150–62. doi: 10.1016/j.arr.2011.07.001 21802530

[20] Bons N. Microcebus murinus: a useful primate model for human cerebral aging and Alzheimer’s disease? Genes Brain Behav. 2005. 10.1111/j.1601-183X.2005.00149.x 16507003

[21] Lührs M-L, Dammhahn M, Kappeler PM, Fichtel C. Spatial memory in the grey mouse lemur (Microcebus murinus). Anim Cogn. 2009;12(4):599–609. doi: 10.1007/s10071-009-0219-y 19263100 PMC2698973

[22] Ho CLA, Zimmermann R, Flórez Weidinger JD, Prsa M, Schottdorf M, Merlin S, et al. Orientation Preference Maps in Microcebus murinus Reveal Size-Invariant Design Principles in Primate Visual Cortex. Curr Biol. 2021;31(4):733–741.e7. doi: 10.1016/j.cub.2020.11.027 33275889 PMC9026768

[23] Jeon H, Kim J, Kim J, Choi YK, Ho CLA, Pifferi F, et al. eLemur: A cellular-resolution 3D atlas of the mouse lemur brain. Proc Natl Acad Sci U S A. 2024;121(50):e2413687121. doi: 10.1073/pnas.2413687121 39630862 PMC11648901

[24] Ezran C. The mouse lemur, a genetic model organism for primate biology, behavior, and health. Genetics. 2017. 10.1534/genetics.116.199448 PMC5499178 28592502

[25] Chaudron Y, Pifferi F, Aujard F. Overview of age-related changes in psychomotor and cognitive functions in a prosimian primate, the gray mouse lemur (Microcebus murinus): Recent advances in risk factors and antiaging interventions. Am J Primatol. 2021;83(11):e23337. doi: 10.1002/ajp.23337 34706117

[26] Faul F, Erdfelder E, Buchner A, Lang A-G. Statistical power analyses using G*Power 3.1: tests for correlation and regression analyses. Behav Res Methods. 2009;41(4):1149–60. doi: 10.3758/BRM.41.4.1149 19897823

[27] Picq J-L, Villain N, Gary C, Pifferi F, Dhenain M. Jumping Stand Apparatus Reveals Rapidly Specific Age-Related Cognitive Impairments in Mouse Lemur Primates. PLoS One. 2015;10(12):e0146238. doi: 10.1371/journal.pone.0146238 26716699 PMC4696676

[28] Languille S, Liévin-Bazin A, Picq J-L, Louis C, Dix S, De Barry J, et al. Deficits of psychomotor and mnesic functions across aging in mouse lemur primates. Front Behav Neurosci. 2015;8:446. doi: 10.3389/fnbeh.2014.00446 25620921 PMC4288241

[29] Masseguin C, LePanse S, Corman B, Verbavatz JM, Gabrion J. Aging affects choroidal proteins involved in CSF production in Sprague-Dawley rats. Neurobiol Aging. 2005;26(6):917–27. doi: 10.1016/j.neurobiolaging.2004.07.013 15718051

[30] Youh R. Evidence for reduced choroid plexus volume in the aged brain. Fluids and barriers of the CNS. 2025. 10.1186/s12987-025-00716-y PMC12506263 41057861

[31] Ge SX, Jung D, Yao R. ShinyGO: a graphical gene-set enrichment tool for animals and plants. Bioinformatics. 2020;36(8):2628–9. doi: 10.1093/bioinformatics/btz931 31882993 PMC7178415

[32] Riaz B, Islam SMS, Ryu HM, Sohn S. CD83 Regulates the Immune Responses in Inflammatory Disorders. Int J Mol Sci. 2023;24(3):2831. doi: 10.3390/ijms24032831 36769151 PMC9917562

[33] Mottahedin A. Choroid plexus transcriptome and ultrastructure analysis reveals a TLR2-specific chemotaxis signature and cytoskeleton remodeling in leukocyte trafficking. Brain, Behavior, and Immunity. 2020. 10.1016/j.bbi.2019.02.004 PMC6591031 30822467

[34] Dolgodilina E. Choroid plexus LAT2 and SNAT3 as partners in CSF amino acid homeostasis maintenance. Fluids and barriers of the CNS. 2020. 10.1186/s12987-020-0178-x PMC7014681 32046769

[35] Ji J-D, Park-Min K-H, Ivashkiv LB. Expression and function of semaphorin 3A and its receptors in human monocyte-derived macrophages. Hum Immunol. 2009;70(4):211–7. doi: 10.1016/j.humimm.2009.01.026 19480842 PMC4811352

[36] Limoni G. Modelling and Refining Neuronal Circuits with Guidance Cues: Involvement of Semaphorins. Int J Mol Sci. 2021;22(11):6111. doi: 10.3390/ijms22116111 34204060 PMC8201269

[37] Kiseleva EP, Rutto KV. Semaphorin 3A in the Immune System: Twenty Years of Study. Biochemistry (Mosc). 2022;87(7):640–57. doi: 10.1134/S0006297922070069 36154881 PMC9282903

[38] Roepke TK, Kanda VA, Purtell K, King EC, Lerner DJ, Abbott GW. KCNE2 forms potassium channels with KCNA3 and KCNQ1 in the choroid plexus epithelium. FASEB J. 2011;25(12):4264–73. doi: 10.1096/fj.11-187609 21859894 PMC3236621

[39] M Y S K. Hydrocephalus and aquaporins: the role of aquaporin-1. Acta Neurochirurgica Supplement. 2012. 10.1007/978-3-7091-0923-6_11 22116423

[40] Hua Y, Ying X, Qian Y, Liu H, Lan Y, Xie A, et al. Physiological and pathological impact of AQP1 knockout in mice. Biosci Rep. 2019;39(5):BSR20182303. doi: 10.1042/BSR20182303 31023968 PMC6522737

[41] Xu H, Fame RM, Sadegh C, Sutin J, Naranjo C, Della S, et al. Choroid plexus NKCC1 mediates cerebrospinal fluid clearance during mouse early postnatal development. Nat Commun. 2021;12(1):447. doi: 10.1038/s41467-020-20666-3 33469018 PMC7815709

[42] MacAulay N, Toft-Bertelsen TL. Dual function of the choroid plexus: Cerebrospinal fluid production and control of brain ion homeostasis. Cell Calcium. 2023;116:102797. doi: 10.1016/j.ceca.2023.102797 37801806

[43] Corino C, Aimo A, Luigetti M, Ciccone L, Ferrari Chen YF, Panichella G, et al. Tetrameric Transthyretin as a Protective Factor Against Alzheimer’s Disease. Mol Neurobiol. 2025;62(3):2945–54. doi: 10.1007/s12035-024-04442-8 39192044 PMC11790689

[44] Buxbaum N. The role of CSF transthyretin in human Alzheimer’s disease: offense, defense, or not so innocent bystander. J Integr Neurosci. 2023. 10.31083/j.jin2206158 38176942

[45] Boulinguez-Ambroise G, Herrel A, Pouydebat E. Ontogeny of locomotion in mouse lemurs: Implications for primate evolution. J Hum Evol. 2020;142:102732. doi: 10.1016/j.jhevol.2019.102732 32172006

[46] Brazidec ML. How aging affects grasping behavior and pull strength in captive gray mouse lemurs (Microcebus murinus). Int J Primatol. 2017.

[47] Canu M-H, Fourneau J, Coq J-O, Dannhoffer L, Cieniewski-Bernard C, Stevens L, et al. Interplay between hypoactivity, muscle properties and motor command: How to escape the vicious deconditioning circle? Ann Phys Rehabil Med. 2019;62(2):122–7. doi: 10.1016/j.rehab.2018.09.009 30394346

[48] Young JW, Shapiro LJ. Developments in development: What have we learned from primate locomotor ontogeny? Am J Phys Anthropol. 2018;165 Suppl 65:37–71. doi: 10.1002/ajpa.23388 29380887

[49] Lacreuse A, Raz N, Schmidtke D, Hopkins WD, Herndon JG. Age-related decline in executive function as a hallmark of cognitive ageing in primates: an overview of cognitive and neurobiological studies. Philos Trans R Soc Lond B Biol Sci. 2020;375(1811):20190618. doi: 10.1098/rstb.2019.0618 32951543 PMC7540957

[50] de Witte A. Preserved cerebellar functions despite structural degeneration in older adults. Neuroscience. 2025.

[51] Bertrand A. Micro-MRI Study of Cerebral Aging: Ex Vivo Detection of Hippocampal Subfield Reorganization. Microhemorrhages and Amyloid Plaques in Mouse Lemur Primates. PLoS One. 2013. 10.1371/journal.pone.0056593 PMC3584101 23460806

[52] Rathke E-M, Fischer J. Differential ageing trajectories in motivation, inhibitory control and cognitive flexibility in Barbary macaques (Macaca sylvanus). Philos Trans R Soc Lond B Biol Sci. 2020;375(1811):20190617. doi: 10.1098/rstb.2019.0617 32951548 PMC7540953

[53] Ma S, Ji Z, Zhang B, Geng L, Cai Y, Nie C, et al. Spatial transcriptomic landscape unveils immunoglobin-associated senescence as a hallmark of aging. Cell. 2024;187(24):7025-7044.e34. doi: 10.1016/j.cell.2024.10.019 39500323

[54] Scarpetta V. Morphological and mitochondrial changes in murine choroid plexus epithelial cells during healthy aging. Fluids and barriers of the CNS. 2023. 10.1186/s12987-023-00420-9 PMC10012601 36918889

[55] Lolansen SD, Rostgaard N, Andreassen SN, Simonsen AH, Juhler M, Hasselbalch SG, et al. Elevated CSF inflammatory markers in patients with idiopathic normal pressure hydrocephalus do not promote NKCC1 hyperactivity in rat choroid plexus. Fluids Barriers CNS. 2021;18(1):54. doi: 10.1186/s12987-021-00289-6 34863228 PMC8645122

[56] Deffner F, Gleiser C, Mattheus U, Wagner A, Neckel PH, Fallier-Becker P, et al. Aquaporin-4 expression in the human choroid plexus. Cell Mol Life Sci. 2022;79(2):90. doi: 10.1007/s00018-022-04136-1 35072772 PMC8785037

[57] Duarte C. Age, sex hormones, and circadian rhythm regulate the expression of amyloid-beta scavengers at the choroid plexus. Int J Mol Sci. 2020. 10.3390/ijms21186813 PMC7554684 32957439

[58] Chen LR. Decrease of transthyretin synthesis at the blood-cerebrospinal fluid barrier of old sheep. J Gerontol. 2005. 10.1093/gerona/60.7.852 16079207

[59] Sadanandan J. Regulation of trophic factors in the choroid plexus of aged mice. Res Sq. 2024.

[60] Serot JM, Christmann D, Dubost T, Couturier M. Cerebrospinal fluid transthyretin: aging and late onset Alzheimer’s disease. J Neurol Neurosurg Psychiatry. 1997;63(4):506–8. doi: 10.1136/jnnp.63.4.506 9343132 PMC2169793

[61] Spector R, Johanson CE. Sustained choroid plexus function in human elderly and Alzheimer’s disease patients. Fluids Barriers CNS. 2013;10(1):28. doi: 10.1186/2045-8118-10-28 24059870 PMC3849253

