# Supplementary material for: Age-related molecular and functional remodeling of the choroid plexus in the gray mouse lemur
Source: PLoS One. 2026 Aug 31;21(8):e0357233. doi: 10.1371/journal.pone.0357233 (PMC13528958; doi:10.1371/journal.pone.0357233)
Supplement: Supplementary file 1 [file pone.0357233.s007.pdf]

We sincerely thank the reviewer for the careful evaluation of our manuscript and for the positive comments regarding the novelty, rigor, and overall quality of our work. We appreciate the constructive suggestions, which have helped us improve the manuscript. Our responses to each comment are provided below.

Comment 1: Sample size. The number of animals is not equal in different age groups. It is clear that the overall number of such difficult-to-obtain animals as lemurs in lab is limited by natural reasons. However, there still exists a problem of rationale for the difference of sample size. Please, add the appropriate calculations into "Materials and methods".

Response: As correctly noted by the reviewer, the availability of aged gray mouse lemurs is inherently limited due to the restricted size of breeding colonies and the natural age distribution of the population. Consequently, unequal group sizes are common in aging studies using this non-human primate model. However, sample size was determined based on an *a priori* sensitivity analysis performed with G*Power 3.1 (PMID: 19897823) for a one-way ANOVA (three age groups), assuming a significance level of $\alpha = 0.05$ and a statistical power of 80%. With the available sample sizes (RNA sequencing analysis, $n = 16$; histological and immunofluorescence analyses, $n = 12$), the study was powered to detect large effect sizes (Cohen's $f \geq 0.87$ and ≥ 1.07, respectively). These sample sizes were considered appropriate given the ethical and practical constraints associated with studies involving captive non-human primates. The corresponding text has been added to the revised manuscript.

Comment 2: Physiological tests are well presented, but histological study is very limited. Exactly, the morphology of tissues near choroid plexus can only be seen from minor images stained by hematoxylin eosin. Why not using any additional staining (at least Nissl) to let the reader understand the morphological side better? Then, histoscans are given in a very small size. May you please add full-size histoscans to supplementary files? IF unveils no queries but the classical histology is too poor.

Response: Our primary objective was to characterize age-related changes in the choroid plexus rather than the surrounding brain parenchyma. Consequently, we focused our histological analyses on hematoxylin and eosin staining (instead of Nissl staining) to evaluate the overall tissue architecture. Following the reviewer's suggestion, we have also included high-resolution full-slide histological images in the Supplementary Material to allow readers to better appreciate the overall morphology of non-human primate brains (now Supplemental figure 1) and we provided a detailed anatomical analysis of adjacent tissues (cortical and hippocampal regions) (now Supplemental figure 2).

Comment 3: May you please specify discussion more on species-related features that may affect the motor results? Readers widely know common animal models but motor tests for lemurs should be explained in more associations with their mode of living, especially when trying to extrapolate into humans. That would add rigor.

Response: We have expanded the discussion to better address the ecological and behavioral characteristics of the gray mouse lemur relevant to motor assessment, including its arboreal lifestyle and reliance on balance, coordination, and grasping. We also clarify the relevance of these tests to age-related functional decline and the need for caution when extrapolating species-specific findings to humans.

Comment 4: Most keywords simply double the title. May be enlarge the section? The work merits to be cited well in future. I would advice to add may be "aquaporin" and "TTR" revealing interesting data; why not 'non-human primates', too?

Response: We appreciate this suggestion. We have revised the list of keywords to improve discoverability of the manuscript. In addition to the original terms, we have included aquaporin, transthyretin (TTR), and non-human primates, as suggested by the reviewer.

Comment 5: The work would gain a lot from a more exact conclusion. For now, it is absent even in abstract, as well as at the end of discussion. You simply outline the most interesting results, but what's next? Please clearly emphasize what do you conclude from testing the hypothesis to make your study sound direct and clear.

Response: We thank the reviewer for this important comment. We have revised the manuscript to make the hypothesis and its outcome more explicit. First, we now explicitly state in the Introduction that we hypothesized that aging would be associated with structural and functional deterioration of the CP, and that these changes would be associated with age-related behavioral decline. We also added a dedicated “Conclusions and perspectives” paragraph at the end of the Discussion. This paragraph explicitly states that CP aging in the mouse lemur is better characterized by selective vulnerability and functional reorganization than by uniform degeneration. Finally, we revised the Abstract to provide a direct conclusion.
