# Supplementary material for: Age-related molecular and functional remodeling of the choroid plexus in the gray mouse lemur
Source: PLoS One. 2026 Aug 31;21(8):e0357233. doi: 10.1371/journal.pone.0357233 (PMC13528958; doi:10.1371/journal.pone.0357233)
Supplement: Peer review history

# Peer review: Decision Letter 0

**DOI:** 10.1371/journal.pone.0357233.r001

**Authors:** Serkan Erdoğan

20 May 2026

Dear Dr. Laurent,

Thank you for submitting your manuscript to PLOS ONE. After careful consideration, we feel that it has merit but does not fully meet PLOS ONE’s publication criteria as it currently stands. Therefore, we invite you to submit a revised version of the manuscript that addresses the points raised during the review process.

==============================

Please carefully follow the referees' suggestions and corrections.

==============================

Please submit your revised manuscript by Jul 03 2026 11:59PM. If you will need more time than this to complete your revisions, please reply to this message or contact the journal office at plosone@plos.org. When you're ready to submit your revision, log on to https://www.editorialmanager.com/pone/ and select the 'Submissions Needing Revision' folder to locate your manuscript file.

- A letter that responds to each point raised by the academic editor and reviewer(s). You should upload this letter as a separate file labeled 'Response to Reviewers'.
- A marked-up copy of your manuscript that highlights changes made to the original version. You should upload this as a separate file labeled 'Revised Manuscript with Track Changes'.
- An unmarked version of your revised paper without tracked changes. You should upload this as a separate file labeled 'Manuscript'.

If you would like to make changes to your financial disclosure, please include your updated statement in your cover letter. Guidelines for resubmitting your figure files are available below the reviewer comments at the end of this letter.

If applicable, we recommend that you deposit your laboratory protocols in protocols.io to enhance the reproducibility of your results. Protocols.io assigns your protocol its own identifier (DOI) so that it can be cited independently in the future. For instructions see: https://journals.plos.org/plosone/s/submission-guidelines#loc-laboratory-protocols. Additionally, PLOS ONE offers an option for publishing peer-reviewed Lab Protocol articles, which describe protocols hosted on protocols.io. Read more information on sharing protocols at https://plos.org/protocols?utm_medium=editorial-email&utm_source=authorletters&utm_campaign=protocols.

As the corresponding author, your ORCID iD is verified in the submission system and will appear in the published article. PLOS supports the use of ORCID, and we encourage all coauthors to register for an ORCID iD and use it as well. Please encourage your coauthors to verify their ORCID iD within the submission system before final acceptance, as unverified ORCID iDs will not appear in the published article. *Only*  the individual author can complete the verification step; PLOS staff *cannot*  verify ORCID iDs on behalf of authors.

We look forward to receiving your revised manuscript.

Kind regards,

Serkan Erdoğan, Ph.D.

Academic Editor

PLOS One

Journal requirements:

When submitting your revision, we need you to address these additional requirements.

1. Please ensure that your manuscript meets PLOS ONE's style requirements, including those for file naming. The PLOS ONE style templates can be found at

https://journals.plos.org/plosone/s/file?id=wjVg/PLOSOne_formatting_sample_main_body.pdf and

https://journals.plos.org/plosone/s/file?id=ba62/PLOSOne_formatting_sample_title_authors_affiliations.pdf

2. Please note that your Data Availability Statement is currently missing a direct link to access each database. If your manuscript is accepted for publication, you will be asked to provide these details on a very short timeline. We therefore suggest that you provide this information now, though we will not hold up the peer review process if you are unable.

If the reviewer comments include a recommendation to cite specific previously published works, please review and evaluate these publications to determine whether they are relevant and should be cited. There is no requirement to cite these works unless the editor has indicated otherwise.

Please review your reference list to ensure that it is complete and correct. If you have cited papers that have been retracted, please include the rationale for doing so in the manuscript text, or remove these references and replace them with relevant current references. Any changes to the reference list should be mentioned in the rebuttal letter that accompanies your revised manuscript. If you need to cite a retracted article, indicate the article’s retracted status in the References list and also include a citation and full reference for the retraction notice.

[Note: HTML markup is below. Please do not edit.]

Reviewers' comments:

Reviewer's Responses to Questions

**Comments to the Author**

1. Is the manuscript technically sound, and do the data support the conclusions?

Reviewer #1: Yes

**********

2. Has the statistical analysis been performed appropriately and rigorously?

Reviewer #1: Yes

**********

3. Have the authors made all data underlying the findings in their manuscript fully available?

The PLOS Data policy

Reviewer #1: No

**********

4. Is the manuscript presented in an intelligible fashion and written in standard English?

Reviewer #1: Yes

**********

Reviewer #1: The manuscript reports novel data about studies of aging in primate brain, exactly in the choroid plexus of Microcebus murinus. Authors hypothesize whether there exist a clear physiological and histological evidence of choroid plexus-associated aging in these animals. The overall concept of study is rigorous, interesting, and even fascinating. The narration is well understandable. Some minor points, although, absolutely should be improved to let this work into publication.

1. Sample size. The number of animals is not equal in different age groups. It is clear that the overall number of such difficult-to-obtain animals as lemurs in lab is limited by natural reasons. However, there still exists a problem of rationale for the difference of sample size. Please, add the appropriate calculations into "Materials and methods".

2. Physiological tests are well presented, but histological study is very limited. Exactly, the morphology of tissues near choroid plexus can only be seen from minor images stained by hematoxylin eosin. Why not using any additional staining (at least Nissl) to let the reader understand the morphological side better? Then, histoscans are given in a very small size. May you please add full-size histoscans to supplementary files? IF unveils no queries but the classical histology is too poor.

3. May you please specify discussion more on species-related features that may affect the motor results? Readers widely know common animal models but motor tests for lemurs should be explained in more associations with their mode of living, especially when trying to extrapolate into humans. That would add rigor.

4. Most keywords simply double the title. May be enlarge the section? The work merits to be cited well in future. I would advice to add may be "aquaporin" and "TTR" revealing interesting data; why not 'non-human primates', too?

5. The work would gain a lot from a more exact conclusion. For now, it is absent even in abstract, as well as at the end of discussion. You simply outline the most interesting results, but what's next? Please clearly emphasize what do you conclude from testing the hypothesis to make your study sound direct and clear.

**********

what does this mean?). If published, this will include your full peer review and any attached files.

If you choose “no”, your identity will remain anonymous but your review may still be made public.

**Do you want your identity to be public for this peer review?**  For information about this choice, including consent withdrawal, please see our Privacy Policy

Reviewer #1: No

**********

[NOTE: If reviewer comments were submitted as an attachment file, they will be attached to this email and accessible via the submission site. Please log into your account, locate the manuscript record, and check for the action link "View Attachments". If this link does not appear, there are no attachment files.]

To ensure your figures meet our technical requirements, please review our figure guidelines: https://journals.plos.org/plosone/s/figures

You may also use PLOS’s free figure tool, NAAS, to help you prepare publication quality figures: https://journals.plos.org/plosone/s/figures#loc-tools-for-figure-preparation.

NAAS will assess whether your figures meet our technical requirements by comparing each figure against our figure specifications.

# Author comment: Author response to Decision Letter 1

**DOI:** 10.1371/journal.pone.0357233.r002

11 Aug 2026

We sincerely thank the reviewer for the careful evaluation of our manuscript and for the positive comments regarding the novelty, rigor, and overall quality of our work. We appreciate the constructive suggestions, which have helped us improve the manuscript. Our responses to each comment are provided below.

Comment 1: Sample size. The number of animals is not equal in different age groups. It is clear that the overall number of such difficult-to-obtain animals as lemurs in lab is limited by natural reasons. However, there still exists a problem of rationale for the difference of sample size. Please, add the appropriate calculations into "Materials and methods".

Response: As correctly noted by the reviewer, the availability of aged gray mouse lemurs is inherently limited due to the restricted size of breeding colonies and the natural age distribution of the population. Consequently, unequal group sizes are common in aging studies using this non-human primate model. However, sample size was determined based on an a priori sensitivity analysis performed with G*Power 3.1 (PMID: 19897823) for a one-way ANOVA (three age groups), assuming a significance level of α = 0.05 and a statistical power of 80%. With the available sample sizes (RNA sequencing analysis, n = 16; histological and immunofluorescence analyses, n = 12), the study was powered to detect large effect sizes (Cohen's f ≥ 0.87 and ≥ 1.07, respectively). These sample sizes were considered appropriate given the ethical and practical constraints associated with studies involving captive non-human primates. The corresponding text has been added to the revised manuscript.

Comment 2: Physiological tests are well presented, but histological study is very limited. Exactly, the morphology of tissues near choroid plexus can only be seen from minor images stained by hematoxylin eosin. Why not using any additional staining (at least Nissl) to let the reader understand the morphological side better? Then, histoscans are given in a very small size. May you please add full-size histoscans to supplementary files? IF unveils no queries but the classical histology is too poor.

Response: Our primary objective was to characterize age-related changes in the choroid plexus rather than the surrounding brain parenchyma. Consequently, we focused our histological analyses on hematoxylin and eosin staining (instead of Nissl staining) to evaluate the overall tissue architecture. Following the reviewer's suggestion, we have also included high-resolution full-slide histological images in the Supplementary Material to allow readers to better appreciate the overall morphology of non-human primate brains (now Supplemental figure 1) and we provided a detailed anatomical analysis of adjacent tissues (cortical and hippocampal regions) (now Supplemental figure 2).

Comment 3: May you please specify discussion more on species-related features that may affect the motor results? Readers widely know common animal models but motor tests for lemurs should be explained in more associations with their mode of living, especially when trying to extrapolate into humans. That would add rigor.

Response: We have expanded the discussion to better address the ecological and behavioral characteristics of the gray mouse lemur relevant to motor assessment, including its arboreal lifestyle and reliance on balance, coordination, and grasping. We also clarify the relevance of these tests to age-related functional decline and the need for caution when extrapolating species-specific findings to humans.

Comment 4: Most keywords simply double the title. May be enlarge the section? The work merits to be cited well in future. I would advice to add may be "aquaporin" and "TTR" revealing interesting data; why not 'non-human primates', too?

Response: We appreciate this suggestion. We have revised the list of keywords to improve discoverability of the manuscript. In addition to the original terms, we have included aquaporin, transthyretin (TTR), and non-human primates, as suggested by the reviewer.

Comment 5: The work would gain a lot from a more exact conclusion. For now, it is absent even in abstract, as well as at the end of discussion. You simply outline the most interesting results, but what's next? Please clearly emphasize what do you conclude from testing the hypothesis to make your study sound direct and clear.

Response: We thank the reviewer for this important comment. We have revised the manuscript to make the hypothesis and its outcome more explicit. First, we now explicitly state in the Introduction that we hypothesized that aging would be associated with structural and functional deterioration of the CP, and that these changes would be associated with age-related behavioral decline. We also added a dedicated “Conclusions and perspectives” paragraph at the end of the Discussion. This paragraph explicitly states that CP aging in the mouse lemur is better characterized by selective vulnerability and functional reorganization than by uniform degeneration. Finally, we revised the Abstract to provide a direct conclusion.

Attachment Submitted filename: Response to Reviewers.pdf

# Editor report: Decision Letter 1

**DOI:** 10.1371/journal.pone.0357233.r003

**Authors:** Serkan Erdoğan

16 Aug 2026

Age-related molecular and functional remodeling of the choroid plexus in the gray mouse lemur

PONE-D-26-06796R1

Dear Dr. Laurent,

We’re pleased to inform you that your manuscript has been judged scientifically suitable for publication and will be formally accepted for publication once it meets all outstanding technical requirements.

Within one week, you’ll receive an e-mail detailing the required amendments. When these have been addressed, you’ll receive a formal acceptance letter and your manuscript will be scheduled for publication.

An invoice will be generated when your article is formally accepted. Please note, if your institution has a publishing partnership with PLOS and your article meets the relevant criteria, all or part of your publication costs will be covered. Please make sure your user information is up-to-date by logging into Editorial Manager at Editorial Manager® and clicking the ‘Update My Information' link at the top of the page. For questions related to billing, please contact billing support.

If your institution or institutions have a press office, please notify them about your upcoming paper to help maximize its impact. If they’ll be preparing press materials, please inform our press team as soon as possible -- no later than 48 hours after receiving the formal acceptance. Your manuscript will remain under strict press embargo until 2 pm Eastern Time on the date of publication. For more information, please contact onepress@plos.org.

Kind regards,

Serkan Erdoğan, Prof. Dr.

Academic Editor

PLOS One

# Editor report: Acceptance letter

**DOI:** 10.1371/journal.pone.0357233.r004

**Authors:** Serkan Erdoğan

PONE-D-26-06796R1

PLOS One

Dear Dr. Laurent,

I'm pleased to inform you that your manuscript has been deemed suitable for publication in PLOS One. Congratulations! Your manuscript is now being handed over to our production team.

At this stage, our production department will prepare your paper for publication. This includes ensuring the following:

* All references, tables, and figures are properly cited

* All relevant supporting information is included in the manuscript submission,

* There are no issues that prevent the paper from being properly typeset

You will receive further instructions from the production team, including instructions on how to review your proof when it is ready. Please keep in mind that we are working through a large volume of accepted articles, so please give us a few days to review your paper and let you know the next and final steps.

Lastly, if your institution or institutions have a press office, please let them know about your upcoming paper now to help maximize its impact. If they'll be preparing press materials, please inform our press team within the next 48 hours. Your manuscript will remain under strict press embargo until 2 pm Eastern Time on the date of publication. For more information, please contact onepress@plos.org.

You will receive an invoice from PLOS for your publication fee after your manuscript has reached the completed accept phase. If you receive an email requesting payment before acceptance or for any other service, this may be a phishing scheme. Learn how to identify phishing emails and protect your accounts at https://explore.plos.org/phishing.

If we can help with anything else, please email us at customercare@plos.org.

Thank you for submitting your work to PLOS One and supporting open access.

Kind regards,

PLOS ONE Editorial Office Staff

on behalf of

Prof. Serkan Erdoğan

Academic Editor

PLOS One
